# Venlafaxine antagonizes the noradrenaline-promoted colon cancer progression by inhibiting the norepinephrine transporter

**DOI:** 10.1038/s41420-023-01447-5

**Published:** 2023-05-08

**Authors:** Huahua Zhang, Jiming Han, Jing Zhang, Jiyu Miao, Fang Li, Kaijie Tang, Kai Zhou, Baojun Duan, Wen Li, Jing Cheng, Ying Sun, Ni Hou, Chen Huang

**Affiliations:** 1 Department of Cell Biology and Genetics, School of Basic Medical Sciences, Xi’an Jiaotong University Health Science Center, Xi’an, 710061 China; 2 Medical Research and Experimental Center, Medical College, Yan’an University, Yan’an, 716000 China; 3 Department of Hematology, The Second Affiliated Hospital of Xian Jiaotong University, Xi’an, 710004 China; 4 Department of Medical Oncology of Shaanxi Provincial People’s Hospital, Xi’an, 710068 China; 5 3201 Affiliated Hospital of Medical College of Xi’an Jiaotong University, Hanzhong, 723000 China; 6 Department of Pathology, School of Basic Medical Sciences, Xi’an Jiaotong University Health Science Center, Xi’an, 710061 China; 7 Institute of Genetics and Developmental Biology, Xi’an Jiaotong University, Xi’an, 710061 China; 8 Key Laboratory of Environment and Genes Related to Diseases, Ministry of Education of China, Xi’an Jiaotong University, Xi’an, 710061 China

**Keywords:** Colorectal cancer, Gastrointestinal cancer

## Abstract

Epidemiological studies have demonstrated that the use of antidepressants is associated with a decreased risk of colorectal cancer (CRC); however, the mechanisms behind this association are yet unknown. Adrenergic system contributes to the stress-related tumor progression, with norepinephrine (NE) mainly secreted from adrenergic nerve fibers. Norepinephrine serotonin reuptake inhibitors are successfully used antidepressants. This study demonstrates that a widely used antidepressant venlafaxine (VEN) antagonizes NE-promoted colon cancer in vivo and in vitro. Bioinformatic analysis suggested that NE transporter (NET, SLC6A2), a target of VEN, was closely associated with the prognosis of clinical patients with CRC. In addition, the knockdown of NET antagonized the effect of NE. The NET-protein phosphatase 2 scaffold subunit alpha/phosphorylated Akt/vascular endothelial growth factor pathway partially mediates the antagonizing effect of VEN on NE’s actions in colon cancer cells. These were also confirmed by in vivo experiments. Our findings revealed for the first time that, in addition to its primary function as a transporter, NET also promotes NE-enhanced colon cancer cell proliferation, tumor angiogenesis, and tumor growth. This provides direct experimental and mechanistic evidence for the use of antidepressant VEN in the treatment of CRC and a therapeutic potential for repurposing existing drugs as an anti-cancer approach to improve the prognosis of patients with CRC.

## Introduction

Colorectal cancer (CRC) is the third most common malignancy worldwide, with a 2–4-fold increase in incidence in developing countries over the last two decades [1–3]. Patients with malignant disease frequently experience depression due to various stresses, which can aggravate clinical manifestations and, consequently, affect disease outcome [4, 5]. A growing body of evidence suggests that antidepressant use is associated with a decreased risk of CRC [6–8]. The most effective therapeutic agents used to treat depression are selective serotonin reuptake inhibitors (SSRIs) or norepinephrine serotonin reuptake inhibitors (NSRIs), which inhibit monoamine neurotransmitter reuptake by targeting monoamine transporters [9]. Venlafaxine (VEN) is a widely used NSRI in the treatment of depressive disorders [10], however, the exact influence and mechanisms of VEN on CRC risk and progression are unclear.

Stress mediators, such as circulating neuroendocrine hormones, enhance cancer progression by inhibiting antitumor immune responses. Recent studies have revealed that the neural invasion in tumor stroma modulates the behavior of cancer cells and affects cancer progression [11–14]. CRC is densely innervated by autonomic fibers including adrenergic fibers, which are associated with poor patient survival [15]. The adrenergic system contributes to stress-induced cancer progression. NE is a principal chemical messenger that acts on central noradrenergic and peripheral adrenergic nerve fibers. NE regulates cell proliferation, survival, and tumor progression by activating the beta-adrenergic receptor (β-AR)-cyclic adenosine monophosphate (cAMP)–protein kinase A (PKA) pathway in various cancer cells [16–18]. We previously reported that chronic stress increased serum NE levels and accelerated the development of subcutaneous tumors in mice. In addition, we identified a NE–cAMP responsive element bind protein 1–miR-373 signaling axis that partially mediates NE-promoted colon cancer progression [19, 20]. Since there is an increase in adrenergic burden in patients with CRC, it is worth investigating whether antidepressants affect NE-promoted colon cancer and the underlying mechanisms.

Due to the numerous side effects and low return on investment of newly developed anticancer drugs, researchers and clinicians have adopted established non-cancer drugs that have already been approved for noncancerous diseases, because of the common molecular origins and changes of diverse diseases. This is called “drug repurposing” or “new use for old drugs” [21, 22]. In this study, we first investigated how VEN influenced the effects of NE both in vivo and in vitro. Second, we performed several experiments using bioinformatic analysis, loss/gain-of-function, coimmunoprecipitation, and rescue experiments in vitro and revealed a novel role of NE transporter (NET) in NE-promoted colon cancer that underlies the antagonistic effect of VEN. Finally, we explored these effects and molecular changes in vivo. Our findings provide direct experimental evidence for the use of antidepressants in the treatment of CRC and offer new potential therapeutic or co-therapeutic strategies to improve the prognosis of patients with CRC.

## Results

### Venlafaxine antagonizes the NE-promoted colon cancer in vivo and in vitro

**Fig. 1:**
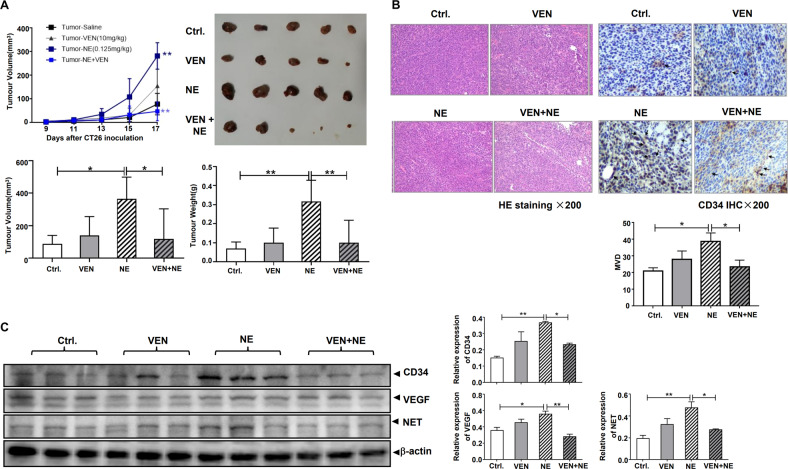
Venlafaxine (VEN) antagonizes norepinephrine (NE)-promoted subcutaneous CT26 tumors’ formation in vivo. After 1-week acclimatization, male BALB/c mice were randomly divided into four groups (*n* = 5): control (saline), NE (0.125 mg/kg), VEN (10 mg/kg), and drug combination [NE (0.125 mg/kg) + VEN (10 mg/kg)], and administered intraperitoneally with each drug or combination every day. One week later, 1 × 10^6^ CT26 cells were injected subcutaneously into the right oxters of the mice. Furthermore, mice of each group were treated with the above drug or drug combination for additional 17 days. **A** The volumes of tumor xenografts were measured and revealed (upper left). On day 17, mice were sacrificed, and tumor xenografts were removed. The gross morphology, tumor volume, and tumor weight of xenografts were detected (upper right, lower left, and lower right). **B** Hematoxylin and eosin (HE) staining and CD34 immunohistochemistry (IHC) analysis of the xenograft sections were performed. Microvessel density (MVD) was counted based on CD34 IHC. **C** The xenograft lysates were collected, and the expressions of CD34, VEGF, and NET were analyzed by western blotting. The band intensities of each protein were normalized by that of β-actin and the average value was obtained from the repetition in each group. Data are expressed as mean ± SD, *P < 0.05, **P < 0.01 in **A**, **B**, **C**.

Initially, we detected the levels of NE in the serum of patients with CRC by ELISA. The amount of serum NE significantly increased as presented in Fig. S1A. Tyrosine hydroxylase (TH) has been used as a biomarker of adrenergic nerves [14]. According to TH IHC data and the RNAseq dataset in TCGA, the expression of TH in cancer tissues was higher than that in adjacent normal colon tissues, which was closely related to the tumor topography and tumor stages of patients with CRC (Fig. S1B, S1C). Therefore, patients with CRC had a high adrenergic load. Since NE is the principal chemical messenger of adrenergic nerve fibers, we intraperitoneally administered NE at various concentrations and observed 0.125 mg/kg of NE was safe for mice, resulting in no apparent weight loss and their continued survival throughout the experiment, which was compliant with animal experiment ethics (Fig. S2A, S2B). On day 17 following CT26 inoculation, the mean weight of the xenografts increased by 6.58 times more than that of the control group, indicating that this treatment caused subcutaneous CT26 tumors formation to occur earlier and faster than the control (Fig. S2C, S2D). Furthermore, we investigated the influence of antidepressant VEN on NE-promoted colon cancer progression. Although VEN alone did not cause any significant changes, VEN treatment [23] inhibited the NE-induced increase of subcutaneous CT26 tumors’ formation. On day 17 following CT26 inoculation, the mean volume and weight of tumors in the NE + VEN group were 25.09% and 28.72% of those in the NE group, respectively (Fig. 1A). HE staining of the xenograft sections revealed that compared with the control, the tumor cells in the NE group had poor differentiation and a rich blood supply. VEN treatment decreased this NE-induced rich blood supply significantly. CD34 is used as a marker of tumor angiogenesis [24]. We performed CD34 IHC analysis and observed that NE induced more CD34 staining while VEN decreased it, with the mean value of MVD in the NE group and NE + VEN group being 38.85 and 23.5, respectively (Fig. 1B). Tumor angiogenesis is an important basis for tumor growth in vivo. In the process of angiogenesis, vascular growth factors play an important regulatory role, and vascular endothelial growth factor (VEGF) can effectively induce mitosis of vascular endothelial cells and cause neovascularization [25–27]. In our experiment, we observed that NE significantly upregulated the expression of CD34 and VEGF, whereas VEN downregulated them in xenograft lysates (Fig. 1C).

**Fig. 2:**
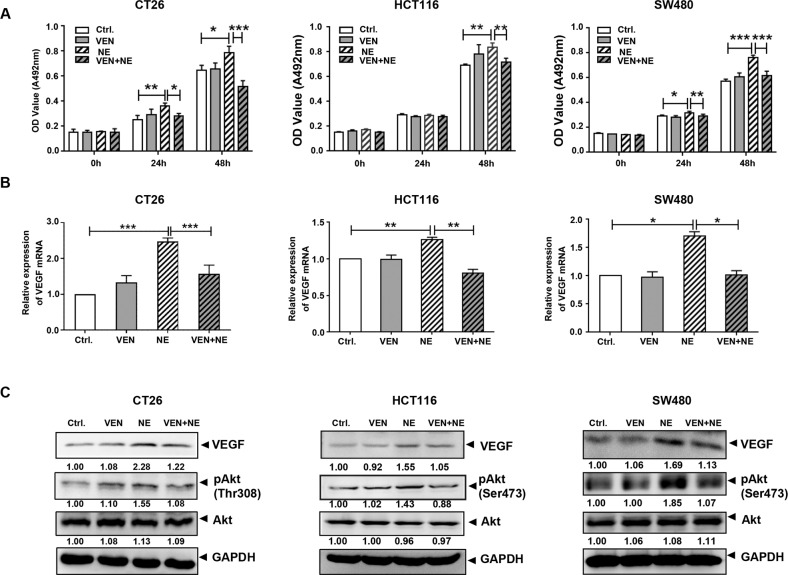
Venlafaxine (VEN) inhibits the norepinephrine (NE)-enhanced cell viability and vascular endothelial growth factor (VEGF) expression in colon cancer cells. Colon cancer cells (CT26, HCT116, and SW480) were treated with control, VEN (10 μM), NE (10 μM), and drug combination [NE (10 μM) + VEN (10 μM)] for 24 h and 48 h separately. **A** The effects of NE/VEN on colon cancer cell proliferation were determined by 3-(4,5-dimethylthiazolyl-2)-2,5-diphenyltetrazolium bromide (MTT) assay. **B** Quantitative reverse transcription-polymerase chain reaction (qRT-PCR) assay was performed and revealed an increase in the VEGF mRNA with NE treatment, while VEN inhibited the NE-increased VEGF mRNA in colon cancer cells. **C** Western blotting was performed and revealed increases in VEGF protein and Akt activation with NE treatment, while VEN inhibited these NE-induced changes in colon cancer cells. The band intensities of VEGF, pAkt, and Akt relative to glyceraldehyde-3-phosphate dehydrogenase (GAPDH) were quantified and normalized to the Ctrl. group. The quantification graphs of normalized band intensities of different replicates were shown in Fig. S8. Data are expressed as mean ± SD, *P < 0.05, **P < 0.01, or ***P < 0.001 in (**A**) and (**B**).

Further, we investigated the influence of VEN on NE-promoted colon cancer in vitro. The results of MTT assays revealed that NE enhanced cell viability in CT26, HCT116, and SW480 cells compared to that of the control; however, VEN inhibited this effect (Fig. 2A). Data from the cell cycle assay revealed that NE increased the cell population in the G2/M phase whereas VEN increased the cell population in the G0/G1 phase. Consistently, the protein level of cyclin E was decreased by VEN treatment (Fig. S3). Since NE significantly increased VEGF levels and blood supply in vivo, we also detected the changes in VEGF in cultured colon cancer cells. NE increased both VEGF mRNA and protein levels, and VEN decreased them in three types of colon cancer cells (Fig. 2B, C). Akt activation has the potential to activate the expression of the *VEGF* gene [28, 29]. As suggested by previous findings, we detected the levels of total Akt and phosphorylated Akt (pAkt) to examine the activation of Akt [30–32]. Although the level of total Akt was not changed in the experiment, the level of pAkt was increased by NE and decreased by VEN (Fig. 2C). These results suggest that VEN antagonized the NE-induced Akt activation, VEGF expression, colon cancer cell proliferation, and tumor growth.

### Venlafaxine antagonizes NE’s effects by inhibiting NET expression

**Fig. 3:**
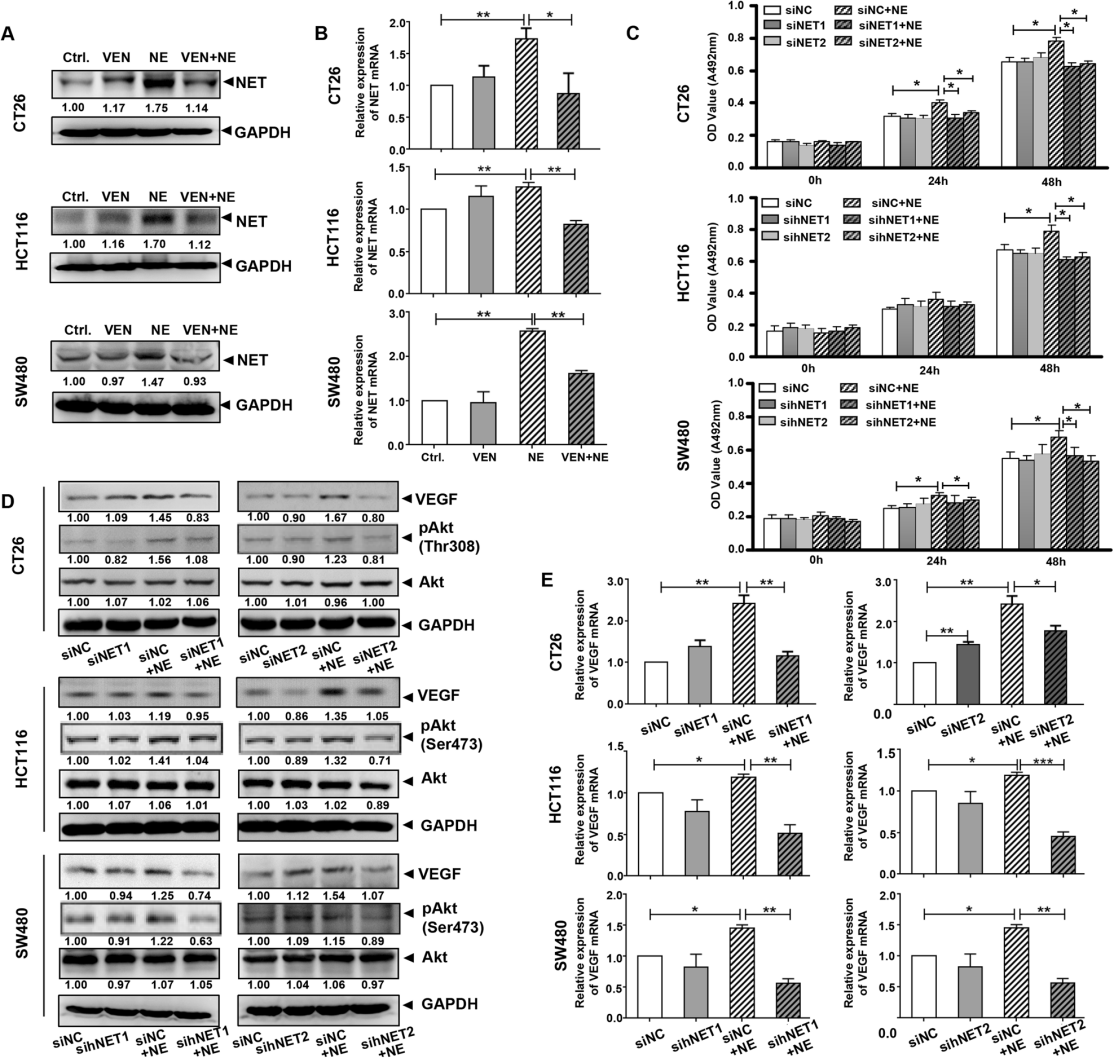
The knockdown of norepinephrine transporter (NET) inhibits the NE-enhanced cell viability and vascular endothelial growth factor (VEGF) expression in colon cancer cells. Colon cancer cells were treated with control, venlafaxine (VEN) (10 μM), NE (10 μM), and drug combination [NE (10 μM) + VEN (10 μM)]. **A** Western blotting and **B** Quantitative reverse transcription-polymerase chain reaction (qRT-PCR) assay were used to detect the changes in NET protein and mRNA. The band intensities of NET relative to glyceraldehyde-3-phosphate dehydrogenase (GAPDH) were quantified and normalized to the Ctrl. group. The quantification graphs of normalized band intensities of different replicates were shown in Fig. S8. **C** Colon cancer cells were transfected with siNET, sihNET, or siNC separately to knock down the expression of NET. After 24 h, they were treated with 10 μM of NE and incubated further for 24 h and 48 h. The effects of NE/siNET on colon cancer cell proliferation were determined by 3-(4,5-dimethylthiazolyl-2)-2,5-diphenyltetrazolium bromide (MTT) assay. **D** Western blotting and **E** qRT-PCR assay were performed and revealed the knockdown of NET inhibited the NE-increased VEGF protein,VEGF mRNA, and Akt activation in colon cancer cells. The band intensities of VEGF, pAkt, and Akt relative to GAPDH were quantified and normalized to the siNC group. The quantification graphs of normalized band intensities of different replicates were shown in Fig. S8. Data are expressed as mean ± SD, *P < 0.05, **P < 0.01, or ***P < 0.001 in (**B**), (**C**) and (**E**).

VEN is an inhibitor of NET and 5-HTT [10]. We analyzed the publicly available CRC gene expression RNAseq datasets from TCGA database and observed that NET expression was closely related to lymphatic metastasis, distant metastasis, and clinical stages of patients with CRC, where 5-HTT was not significantly related to any of them (Fig. S4). Therefore, we observed the changes in NET in our experiment. NE treatment increased both NET protein and mRNA levels in colon cancer cells, which was similar to the previous report [33], however, VEN inhibited this increase (Fig. 3A, B). Consistently, NE in xenograft lysates also upregulated the expression of NET, while VEN downregulated it (Fig. 1C). As an inhibitor of NET, VEN can inhibit the NE-induced NET expression. To determine the role of NET in the effects of NE and VEN, we synthesized four siRNAs (siNET1 and siNET2 specifically targeting mice NET and sihNET1 and sihNET2 specifically targeting human NET), and the negative controls (siNC) (Supplementary Table 2). qRT-PCR and immunoblotting analysis confirmed that transfection of these siRNAs decreased NET protein and mRNA levels (Fig. S5). siNETs and sihNETs inhibited the NE-induced increase of colon cancer cell viability (Fig. 3C). Furthermore, they inhibited the NE-induced increase of pAkt and VEGF (Fig. 3D, E). These data mimicked the antagonizing effect of VEN on NE’s effect. Therefore, NET may contribute to the effects of NE on colon cancer cells, and VEN may antagonize the effects of NE by inhibiting NET.

### NET-PPP2R1A interaction modulates the PPP2R1A protein level and mediates VEN’s antagonizing effect on NE’s effects in colon cancer cells

**Fig. 4:**
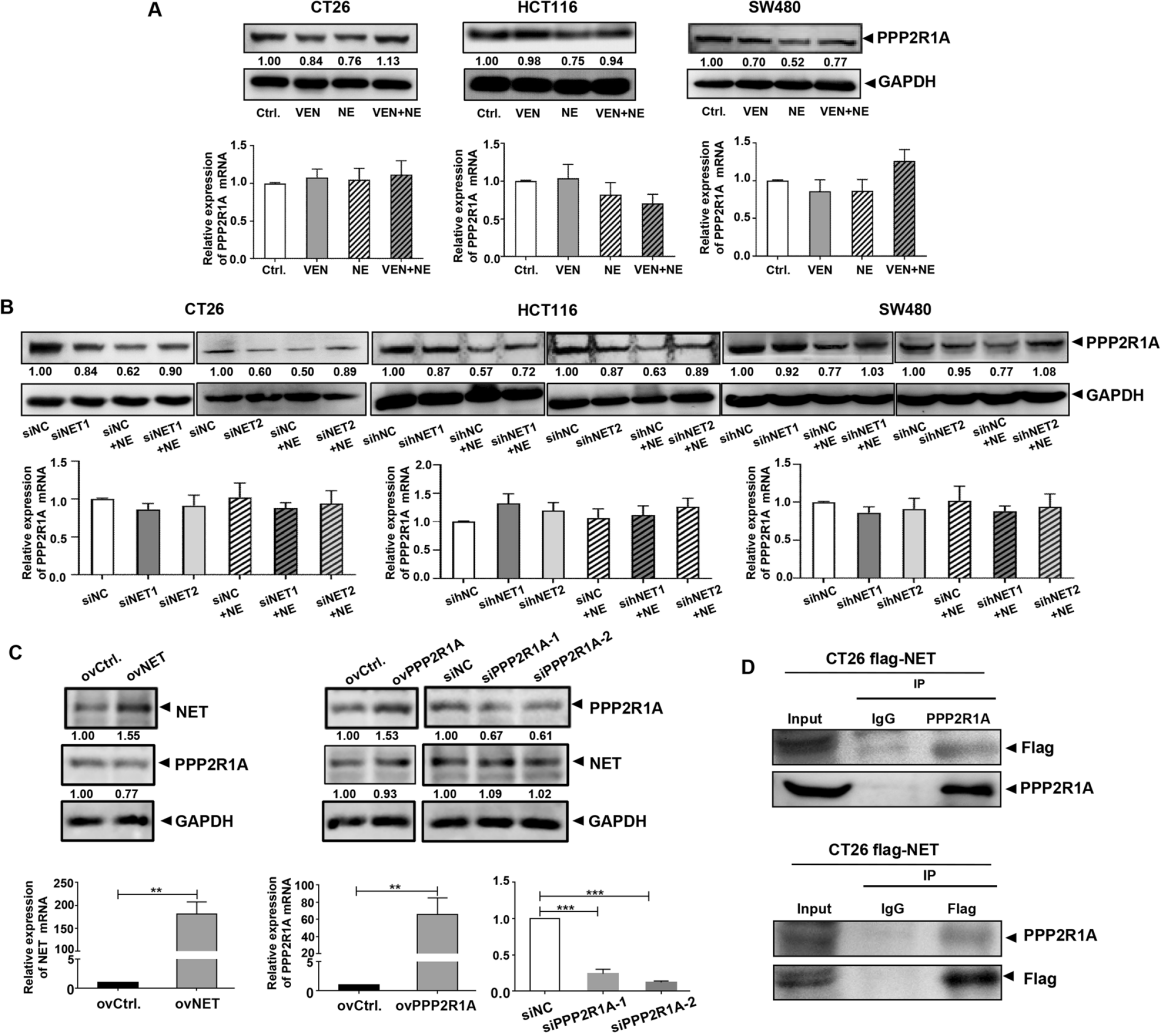
Norepinephrine transporter (NET)-protein phosphatase 2 scaffold subunit alpha (PPP2R1A) interaction modulates PPP2R1A protein level in colon cancer cells. Colon cancer cells were treated with control, venlafaxine (VEN) (10 μM), NE (10 μM), and drug combination [NE (10 μM) + VEN (10 μM)] separately. **A** Western blotting and quantitative reverse transcription-polymerase chain reaction (qRT-PCR) assay were performed and revealed that NE reduced the protein level of PPP2R1A, while VEN increased it; there was no influence on PPP2R1A mRNA levels with NE/VEN treatment. The band intensities of PPP2R1A relative to glyceraldehyde-3-phosphate dehydrogenase (GAPDH) were quantified and normalized to the Ctrl. group. The quantification graphs of normalized band intensities of different replicates were shown in Fig. S8. **B** Colon cancer cells were transfected with siNET, sihNET, or siNC separately. After 24 h, they were treated with 10 μM of NE and incubated further. Western blotting and qRT-PCR assay were performed and revealed that the knockdown of NET caused an increase in NE-reduced PPP2R1A protein expression; there was no influence on PPP2R1A mRNA levels with NE/ siNET treatment. The band intensities of PPP2R1A relative to GAPDH were quantified and normalized to the siNC group. The quantification graphs of normalized band intensities of different replicates were shown in Fig. S8. **C** The plasmids overexpressing NET and PPP2R1A were constructed, and short interfering RNAs against PPP2R1A were synthesized. They were transfected into CT26 cells separately. Western blotting and qRT-PCR were used to detect the changes in NET and PPP2R1A. The band intensities of NET and PPP2R1A relative to GAPDH were quantified and normalized to the ovCtrl./siNC group. The quantification graphs of normalized band intensities of different replicates were shown in Fig. S8. **D** Coimmunoprecipitation (Co-IP) assay was performed and revealed the existence of NET-PPP2R1A interaction. Data are expressed as mean ± SD, **P < 0.01, or ***P < 0.001 in **C**.

Bioinformatic analysis suggested that there was a protein interaction between NET (SLC6A2) and PPP2R1A (Fig. S6). As the scaffold protein of PP2A, the regulation of the PPP2R1A protein level is closely related to the function of PP2A, which plays tumor suppressive roles [34–37]. Therefore, in this experiment, we observed changes in PPP2R1A. NE reduced the PPP2R1A protein level, while VEN increased it. However, no significant changes in PPP2R1A mRNA were detected (Fig. 4A). siNETs and sihNETs also increased the NE-reduced PPP2R1A proteins, without affecting PPP2R1A mRNA levels (Fig. 4B). By transfecting Flag-NET plasmid into CT26 cells, we increased NET expression (Fig. 4C). A coimmunoprecipitation assay was performed, and the results revealed the presence of NET-PPP2R1A interaction (Fig. 4D), which is consistent with previous report [38]. Consistent with the increase of NE-reduced PPP2R1A protein by NET knockdown (Fig. 4B), overexpression of NET decreased the PPP2R1A level. However, PPP2R1A overexpression or knockdown did not cause any corresponding changes in the NET protein (Fig. 4C). These findings suggest that there is a NET-PPP2R1A interaction that modulates PPP2R1A protein levels.

**Fig. 5:**
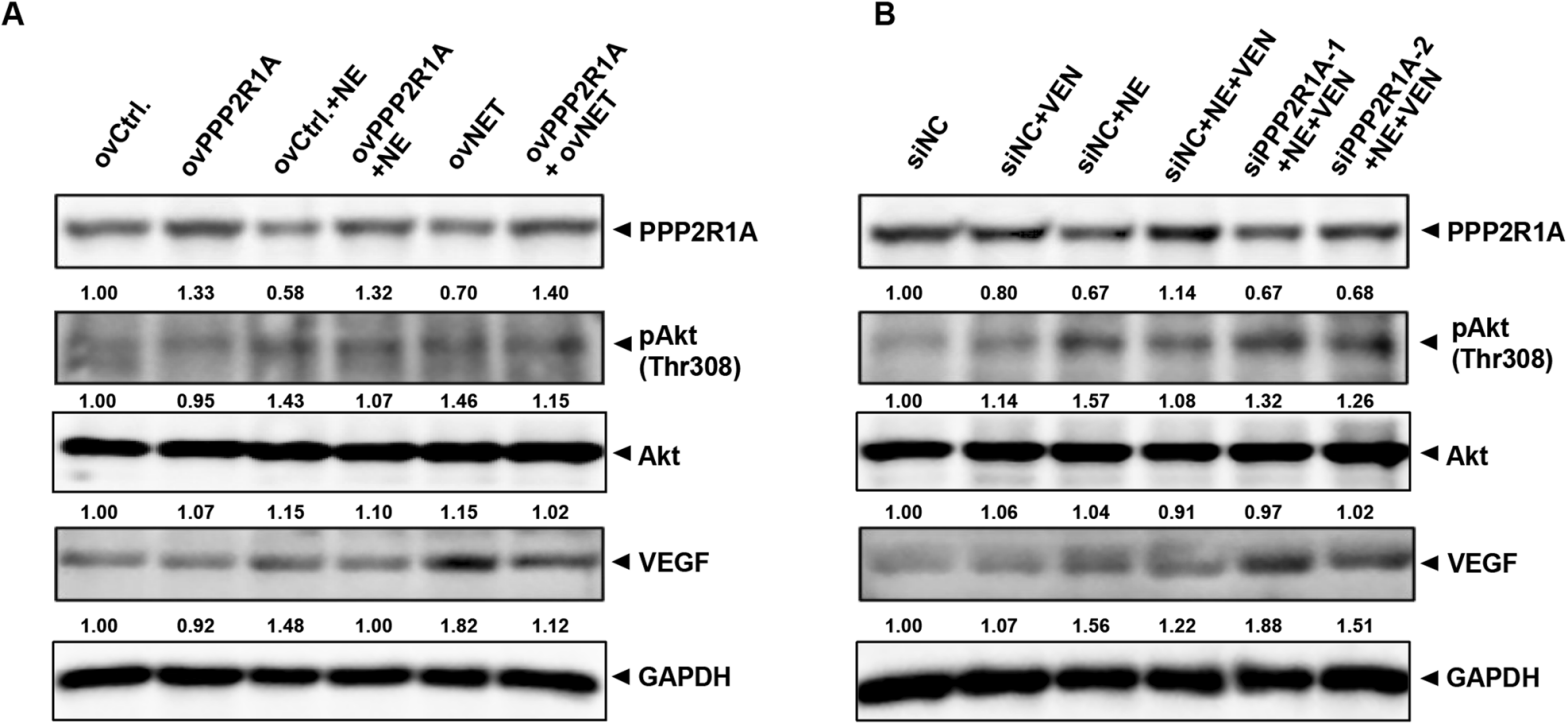
Norepinephrine transporter (NET)-protein phosphatase 2 scaffold subunit alpha (PPP2R1A)-modulated PPP2R1A protein regulates Akt activation and vascular endothelial growth factor (VEGF) expression in colon cancer cells. **A** CT26 cells were transfected with plasmids of ovCtrl., ovPPP2R1A, or ovNET separately. Twenty-four hours later, they were treated with NE or not and incubated further for 48 h. Western blotting was used to detect the changes in PPP2R1A, pAkt, Akt, and VEGF proteins. The quantification graphs of normalized band intensities of different replicates were shown in Fig. S8. **B** CT26 cells were transfected with short interfering RNAs of siNC, siPPP2R1A-1, or siPPP2R1A-2 separately. Twenty-four hours later, they were treated with venlafaxine (VEN), NE, and NE + VEN, or not and incubated further for 48 h. Western blotting was used to detect the changes in PPP2R1A, pAkt, Akt, and VEGF proteins. The band intensities of PPP2R1A, pAkt, Akt, and VEGF relative to glyceraldehyde-3-phosphate dehydrogenase (GAPDH) were quantified and normalized to the ovCtrl./siNC group. The quantification graphs of normalized band intensities of different replicates were shown in Fig. S8. Experiments were repeated three independent times with reproducible results.

Since PPP2R1A is an important component of PP2A that can dephosphorylate Akt and mitogen-activated protein kinase and play tumor-suppressive roles [35–37], we performed a rescue experiment to determine the role of NET-PPP2R1A-modulated PPP2R1A in this study. PPP2R1A overexpression rescued the PPP2R1A decrease, Akt activation, and VEGF increase induced by NE or NET overexpression (Fig. 5A). PPP2R1A was downregulated by siPPP2R1A transfection, which reversed the effects of the combination (NE + VEN) treatment that caused PPP2R1A increase and pAkt and VEGF decrease (Fig. 5B). These data indicate that the NET-PPP2R1A interaction may modulate the level of PPP2R1A protein, which would subsequently mediate the antagonizing effect of VEN on NE’s effects in colon cancer cells.

### The knockdown of NET antagonizes NE-promoted colon cancer with corresponding changes of PPP2R1A, Akt activation, VEGF, and CD34 in tumor xenografts

**Fig. 6:**
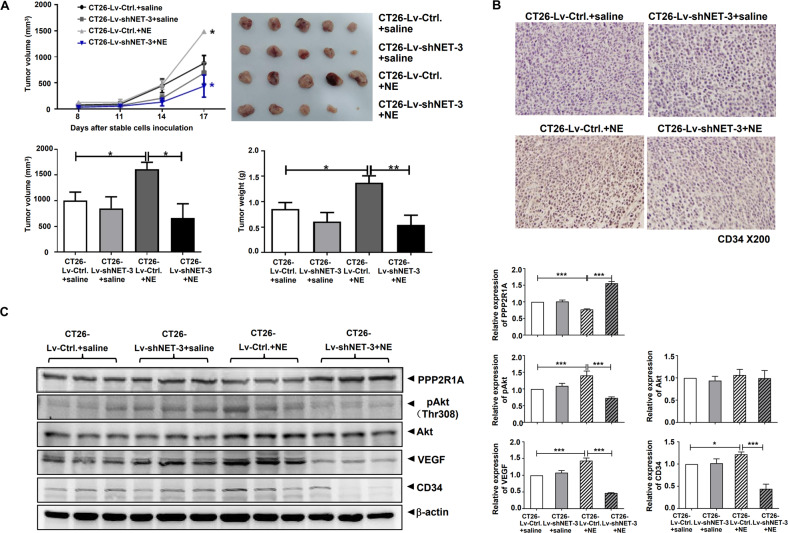
The knockdown of norepinephrine transporter (NET) antagonizes NE-promoted subcutaneous CT26 tumors’ formation in vivo with corresponding changes of protein phosphatase 2 scaffold subunit alpha (PPP2R1A), Akt activation, VEGF, and CD34 in tumor xenografts. **A** Following acclimatization, male BALB/c mice were randomly divided into four groups (*n* = 5): CT26-Lv-shCtrl.+saline, CT26-Lv-shNET-3+saline, CT26-Lv-shCtrl.+NE, and CT26-Lv-shNET-3+NE. Male BALB/c mice were intraperitoneally administered with saline or NE (0.125 mg/kg) daily. One week later, 1 × 10^6^ stable cells (CT26-Lv-shNET-3 or CT26-Lv-shCtrl.) were injected subcutaneously into the mice separately. In addition, mice of each group were treated with saline or NE for 17 days. Volumes of tumor xenografts were measured (upper left). On day 17 following cell inoculation, mice were scarified, and tumor xenografts were removed. The gross morphology of tumors, tumor volume, and tumor weight of each group were detected (upper right, lower left, and lower right). **B** Hematoxylin and eosin (HE) staining of the xenograft sections and CD34 immunohistochemical analysis was performed. **C** Xenograft lysates were collected, and the expression levels of PPP2R1A, pAkt, Akt, VEGF, and CD34 were analyzed by western blotting. The band intensities of each protein were normalized by that of β-actin and the average value was obtained from the repetition in each group. Data are expressed as mean ± SD, *P < 0.05, **P < 0.01, or ***P < 0.001 in **A** and **C**.

Finally, we confirmed the role and regulation of NET in the effect of NE in vivo. After the construction of stable cell lines that demonstrated constantly decreasing NET using lentivirus infection, we selected the CT26-Lv-shNET-3 cell line for the subsequent experiments since the NET protein level was significantly decreased (Fig. S7). The constant decrease of NET significantly slowed down the formation of NE-promoted subcutaneous tumors. On day 17 following inoculation, the CT26-Lv-shNET-3+NE group’s mean tumor volume was 40.99% of that in the CT26-Lv-Ctrl+NE group, and its mean tumor weight was 39.65% of that in CT26-Lv-Ctrl+NE group (Fig. 6A). Xenograft sections from the CT26-Lv-shNET-3+NE group stained less for CD34 than the CT26-Lv-Ctrl+NE group in a CD34 immunohistochemical assay (Fig. 6B). Results from xenograft lysates immunoblotting demonstrated that PPP2R1A protein was decreased and the levels of pAkt, VEGF, and CD34 proteins were increased in CT26-Lv-Ctrl+NE group compared to those in CT26-Lv-Ctrl group; however, these changes were alleviated in the CT26-Lv-shNET-3+NE group (Fig. 6C). These results further suggest that NET participates in NE-promoted colon cancer through the sequential changes of PPP2R1A, Akt activation, VEGF, and CD34 in tumor xenografts. The knockdown of NET can antagonize NE-promoted colon cancer as demonstrated by VEN.

## Discussion

To our knowledge, this is the first study to investigate the anticancer effects of VEN on colon cancer. In addition to alleviating depression, antidepressant use has been reported to be associated with a decreased risk of various cancers, including CRC [6–8]. Previous studies focused on antidepressants regulating tumor progression by improving patients’ mental state and neuroendocrine hormones [11, 12, 39, 40]. Recently local sympathetic innervation of cancer tissues (mainly secreting NE) was reported to promote tumor progression [13–15, 41]. We previously observed that chronic stress increased NE levels in mice serum, accelerated colon cancer formation in mice [19], and caused depression in mice (unpublished data). Since it has been suggested that using the antidepressant fluoxetine may inhibit the growth of colorectal tumors through an anti-promoter effect or direct cytotoxic effect [6], we investigated the effect of the antidepressant VEN on CRC development. Similar to previous studies [12, 15], we detected an increased adrenergic load in patients with CRC and observed that the widely used antidepressant, VEN can antagonize the role of NE in promoting colon cancer cell proliferation, tumor angiogenesis, and tumor growth in vivo and in vitro. This is consistent with epidemiological studies and provides direct evidence that antidepressants can both alleviate cell-extrinsic processes, such as neuroendocrine, immune, and psychological changes and directly act on cancer cells-intrinsic oncogenic events [22] to inhibit the progression of colon cancer.

Tumor stroma constitutes 60–90% of the colon tumor mass [42], and pericryptal colonic stroma surrounding the cryptal bottom has been reported to initiate certain steps in colon tumor development, such as increased proliferation, microvessel formation, VEGF-synthesis, regulation of self-renewal and differentiation of intestinal cells [43]. Tumor angiogenesis is an important basis for tumor growth. Our findings revealed that despite the relatively slight increase in colon cancer cell proliferation caused by NE treatment, NE significantly induced Akt activation and VEGF expression. This is consistent with the previous reports suggesting adrenergic signaling-mediated angiogenesis plays a crucial role in ovarian carcinoma development [12]. VEN treatment can significantly inhibit the NE-induced Akt activation and VEGF expression in vitro and the NE-induced tumor angiogenesis and tumor growth in vivo, with no significant changes by only VEN treatment. Therefore, VEN can antagonize the NE-promoted colon cancer progression, possibly by inhibiting the NE-induced tumor angiogenesis.

VEN is an inhibitor of NET and 5-HTT. Using the public TCGA database, we discovered that compared with the result of 5-HTT, NET was closely associated with lymphatic metastasis, distant metastasis, and clinical stages of patients with CRC. We also discovered that fluoxetine, a selective inhibitor of 5-HTT, had a lower antagonistic effect on the formation of subcutaneous CT26 tumors than that of VEN (unpublished data), although there are other reports demonstrating the antitumor effects of fluoxetine [8, 44]. Furthermore, through loss/gain-of-function, rescue experiment, and in vivo experiment, we discovered for the first time that NET, in addition to performing its primary function of internalizing NE from the synaptic cleft, also promoted NE-enhanced colon cancer cell proliferation, tumor angiogenesis, and tumor growth. This creatively provides evidence for the significance of NET in the diagnosis and treatment of CRC. In addition, VEN antagonized the effects of NE (especially NE-induced tumor angiogenesis) by inhibiting the NE-increased NET expression.

Therefore, what is the signaling pathway mediated by NET to regulate *VEGF* expression? Through bioinformatic analysis, coimmunoprecipitation assay, loss-of-function, and rescue experiment, PPP2R1A, which is a component of the tumor suppressor PP2A, could bind to NET and affect its protein level, dephosphorylate pAkt, and influence its activity of regulating *VEGF* gene expression. VEN antagonized the effect of NE via the NET-PPP2R1A/pAkt/VEGF pathway. The changes in the cell cycle and cyclin E treated with VEN also support PPP2R1A participation since PP2A has been reported to control the G1-S transition in the cell cycle by regulating G1 cyclin stability [45]. Although this pathway cannot completely explain how VEN antagonizes NE to promote colon cancer progression, it may help to elucidate the underlying mechanisms of antidepressants affecting CRC.

This study had a few limitations, which were as follows: Firstly, we observed the NET-PPP2R1A/pAkt/VEGF pathway underlying the VEN’s antagonizing effect. Deep mechanistic insights into NET and PPP2R1A may be lacking in this study and would be worth pursuing in the future. Secondly, the concentration of VEN in our experiment may be different from the therapeutical concentration of VEN in clinical practice. It is necessary to further study the optimal use of VEN, including the concentration and the reaction with traditional chemotherapy drugs, such as 5-FU. Thirdly, using medications that target the nervous system to treat cancer has been proposed as a promising addition to the existing therapy [22, 46]. Tumor cells interact with the neighboring environment, including neuronal tissue during cancer progression. The interaction between adrenergic nerve fibers and CRC progression, and how VEN affects it, deserves further investigation. Nevertheless, since the pharmacokinetic, pharmacodynamic, and toxicity profiles of VEN are well known and could be rapidly translated into clinical studies, it is worth investigating further.

**Fig. 7:**
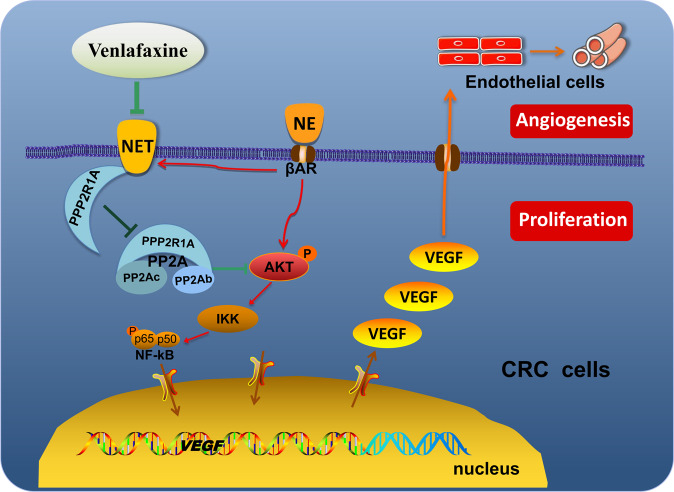
A model for venlafaxine (VEN) antagonizing the NE-promoted colon cancer progression by inhibiting NET. By binding with beta-adrenergic receptor (b-AR), norepinephrine (NE) induces Akt activation, VEGF expression, cell proliferation, angiogenesis, and cancer progression in human colorectal cancer (CRC) cells. NE also increases the expression of NE transporter (NET). Venlafaxine (VEN) can antagonize the above effects of NE by inhibiting the NE-increased NET expression, which is related to the interaction of NET and protein phosphatase 2 scaffold subunit alpha (PPP2R1A).

To the best of our knowledge we reveal for the first time that NET plays a promoting role in NE-enhanced colon cancer cell proliferation, tumor angiogenesis and tumor growth. Its inhibitor antidepressants (such as VEN) can directly act on colon cancer cells and antagonize the role of NE’s effects via NET-PPP2R1A/pAkt/VEGF pathway (Fig. 7). This provides direct experimental support for the use of the antidepressant to treat CRC and a “therapeutic potential for repurposing existing drugs as an anti-cancer approach”. Our data will offer new potential therapeutic or co-therapeutic strategies for improving the prognosis of patients with CRC.

## Materials and methods

### Collection of clinical patients with CRC samples

All samples were collected at the Department of General Surgery of the Second Affiliated Hospital of Xi’an Jiaotong University between September 1, 2019 and September 1, 2021. None of the patients with CRC included in this study had any other tumors or inflammatory colorectal disease, such as colitis or infectious diseases, nor had they received chemotherapy or radiation therapy prior to surgery. The patients who participated in this work expressed informed consent. The experimental protocols were approved specifically by the Biomedical Ethics Committee of Xi’an Jiaotong University Health Science Center (Permission No. 2019-854).

All serum samples were collected, and the serum level of NE was measured by double-antibody sandwich enzyme-linked immunosorbent assay (ELISA) kits (Elisa Biotech, Shanghai, China). The optical density value at 450 nm was measured within 15 min using a microtiter plate reader (FLUOstar Omega, BMG LABTECH GmbH, Germany).

### Antibodies and reagents

Antibodies against NET (mouse) (GeneTex Cat# GTX82626, RRID: AB_11173829) and NET (human) (GeneTex Cat# GTX47102, RRID: AB_11164269) were purchased from GeneTex (GeneTex, USA). Anti-phospho-Akt (Ser473) (Cell Signaling Technology Cat# 4060, RRID: AB_2315049) and Anti-phospho-Akt (Thr308) (Cell Signaling Technology Cat# 13038, RRID: AB_2629447) were obtained from Cell Signaling Technology, Inc. (Beverly, MA, USA). Antibodies against CD31 (Proteintech Cat# 11265-1-AP, RRID: AB_2299349), CD34 (Proteintech Cat# 14486-1-AP, RRID: AB_2228975), vascular endothelial growth factor (VEGF, Proteintech Cat# 19003-1-AP, RRID: AB_2212657), CDK2 (Proteintech Cat# 10122-1-AP, RRID: AB_2078556), cyclin E (Proteintech Cat# 11554-1-AP, RRID: AB_2071066), Akt (Proteintech Cat# 10176-2-AP, RRID: AB_2224574), and protein phosphatase 2 scaffold subunit alpha (PPP2R1A, Proteintech Cat# 15882-1-AP, RRID: AB_2237574) were purchased from Proteintech Group (Wuhan, Hubei, China). Anti-glyceraldehyde-3-phosphate dehydrogenase (Santa Cruz Biotechnology Cat# sc-32233, RRID: AB_627679) and anti-β-actin (Santa Cruz Biotechnology Cat# sc-47778, RRID: AB_626632) were from Santa Cruz Biotechnology, Inc (Santa Cruz, CA, USA). The horseradish peroxidase (HRP)-conjugated IgG anti-rabbit (Jackson ImmunoResearch Labs Cat# 111-035-144, RRID: AB_2307391) and anti-mouse (Jackson ImmunoResearch Labs Cat# 115-035-146, RRID: AB_2307392) secondary antibodies were obtained from Jackson ImmunoResearch Laboratories, Inc (Jackson ImmunoResearch, PA, USA). They are listed in Supplementary Table 1. Norepinephrine (NE) was purchased from Sigma (Sigma, USA), and VEN from Cayman Chemical (Cayman Chemical, USA).

### Constructions of plasmids and siRNAs

GV141 vector containing cDNA of NET, CV129 vector containing cDNA of PPP2R1A and the corresponding control vectors were purchased from GeneChem (Genechem Co. Ltd., Shanghai, China). siRNAs against NET and PPP2R1A were designed and synthesized by GenePharma (Shanghai, China). Scramble siRNA (siNC) was used as the negative control. The sequences are listed in Supplementary Table 2.

### Animal xenograft studies

Male BALB/c mice aged between 4–6 weeks and weighing 20–25 g were obtained from the animal experiment center of Xi’an Jiaotong University. The animal experimental protocols were approved by the Biomedical Ethics Committee of Xi’an Jiaotong University Health Science Center (Permission No. 2019-854).

For the xenograft model, in brief, 1 × 10^6^ CT26 cells (0.1 mL of single-cell suspensions) were administered subcutaneously into the right oxter of the mice. Tumor growth was measured every 3 days using vernier calipers. The tumor size was calculated using the formula: V = 1/2 ab^2^, where a represents the largest tumor diameter and b represents the smallest tumor diameter. On day 17 of CT26 inoculation, the mice were euthanized by cervical dislocation, and tumors were harvested and weighed. A portion of the xenografts was excised and fixed or stored at −80 °C for further analysis.

### Hematoxylin and eosin (HE) staining, immunohistochemistry (IHC), and analysis

For hematoxylin and eosin (HE) staining, tissue sections (5 µm) were deparaffinized using xylene, rehydrated in gradient ethanol, and stained with hematoxylin and eosin according to manufacturer’s instructions. The HE staining results were taken to avoid tumor necrosis and edge photographs as far as possible.

For IHC analysis, tissue sections were deparaffinized, rehydrated, incubated in endogenous peroxidase inhibitor, and blocked with goat serum working solution (all at room temperature). Subsequently, they were incubated with anti-CD34 (Proteintech Group) followed by incubation with a secondary antibody conjugated with HRP. Detection of CD34 distribution was conducted by 3,3’-diaminobenzidine and hematoxylin. Two independent pathologists who were blind to the clinical data of the patients evaluated the IHC photos. The microvessel density (MVD) in each specimen was evaluated by counting anti-CD34 positive microvessels and calculated by the method described by Weidner et al. [47].

### Cell culture and transfection

Human colon cancer cells [HCT116 (RRID: CVCL_0291) and SW480 (RRID: CVCL_0546)] and mouse colon cancer cells (CT26) (RRID: CVCL_7254) were maintained in the Key Laboratory of Environment and Genes Related to Diseases at Xi’an Jiaotong University. The cells were cultured in Rosewell Park Memorial Institute-1640 medium (HyClone, Logan, UT, USA) supplemented with 10% fetal bovine serum (Biological Industries, Beit Haemek, Israel) at 37 °C in a humidified atmosphere of 95% air and 5% CO_2_. Plasmid and siRNAs were transiently transfected into the cells using Lipofectamine 2000 (Invitrogen, Carlsbad, CA, USA), according to the manufacturer’s recommendation.

### Cell proliferation assay and cell cycle assay

Colon cancer cells of each group were treated with the drug (NE/VEN) and incubated for 24, 48, and 72 h following drug treatment. Ten microliters of 3-(4,5-dimethylthiazolyl-2)-2,5-diphenyltetrazolium bromide (MTT) reagent (Sigma, St. Louis, MO, USA) was added. After 4 h incubation, the culture medium was removed, and 100 μL of dimethyl sulfoxide (Sigma, USA) was added. The cell viability was evaluated by measuring the absorbance at 490 nm using the MTT assay FLUO star OPTIMA (BMG LABTECH, Offenburg, Germany).

Colon cancer cells of each group were collected after 24 h of drug treatment. They were washed and fixed with 70% pre-cooled ethanol at 4 °C overnight. After being washed and incubated in 0.1 mg/mL RNase A and 0.05 mg/mL propidium iodide for 30 min at 4 °C in the dark, the cell cycle changes of each group were detected by flow cytometry (FACSort; Becton) within 30 min.

### Quantitative reverse transcription-polymerase chain reaction (qRT-PCR)

Cells were treated as described in each figure legend. After 24 h, the total cellular RNA was extracted using TRIzol reagent (Invitrogen, Carlsbad, CA, USA) according to the manufacturer’s instructions. cDNA was synthesized using the PrimeScript RT reagent kit (Takara, Otsu, Japan) according to the manufacturer’s protocol (Takara, Dalian, China). qRT‑PCR was performed using SYBR Green Master Mix (Takara) on an FTC-3000TM System (Funglyn Biotech Inc., Toronto, Ontario, Canada) according to the manufacturer’s instructions. The 2^−ΔΔCT^ method was used to quantify the relative levels of mRNA. Primers were synthesized by Beijing Qingke Xinye Biotechnology Co., Ltd., and all primers used in this study are presented in Supplementary Table 3.

### Western Blotting

Cells were treated as described in each figure legend. After 48 h, they were lysed in radio-immunoprecipitation assay buffer (Pioneer, Shanghai, China) supplemented with protease and phosphatase inhibitors (Invitrogen). Protein concentration quantification was determined using a bicinchoninic acid protein assay kit (Pierce). Membranes were incubated with primary antibodies overnight at 4 °C. On the second day, they were further incubated with the corresponding anti-rabbit/anti-mouse secondary antibodies (Jackson ImmunoResearch Laboratories, Inc., USA) for 1 h at room temperature. The membrane was incubated with enhanced chemiluminescence (Pierce, Rockford, IL, USA) in the dark for chemiluminescence detection. Luminescent signals were detected and recorded by Syngene GBox (Syngene, Cambridge, UK).

### Bioinformatic analysis

Data on TH, NET, serotonin transporter (5-HTT), tumor topography (T), lymph node metastasis (N), distant metastasis (M), and stage of patients with CRC were downloaded from the Cancer Genome Atlas (TCGA) database (http://xenabrowser.net). One-way analysis of variance (ANOVA) followed by Tamhane’s T2 post hoc test was used to determine the associations between NET, 5-HTT expression level, and clinical characteristics of patients with CRC. Proteins interacting with NETs were searched through the STRING, Protein Interactions (PPI Network) database (http://cn.string-db.org).

### Coimmunoprecipitation (Co-IP) assay

CT26 cells were transfected with NET overexpression vector containing a flag tag using Lipofectamine 2000 (Invitrogen) and were lysed 48 h later with IP lysis buffer. The cell lysates were pre-cleared with protein G agarose beads (Beyotime Biotechnology, Jiangsu, China) for 3 h at 4 °C. Subsequently, equal amounts of sample lysate were incubated with either 1.0 μg of IgG or primary antibodies overnight at 4 °C. The beads were then thrice with pre-cooled phosphate-buffered saline, and the pellets were dissolved into 2× sodium dodecyl-sulfate loading buffer after centrifugation. The protein was analyzed by western blotting with different antibodies.

### Construction of stable cell line

The packaged lentivirus of sh-NET was constructed by GeneChem (Shanghai, China) and named Lv-shNET. The scramble lentivirus (Lv-shCtrl.) was used as a control. They were transfected into CT26 cells separately. Furthermore, stable infected cell lines (CT26-Lv-Ctrl. and CT26-Lv-shNET) were obtained by puromycin screening.

### Statistical Analyses

Each of the experiment were conducted for three or more times. The data were expressed as the mean ± standard deviation (SD) and analyzed with SPSS 18.0 software (SPSS, Inc.). Student’s t-test was used to compare the means between two independent samples, while the comparisons of means between multiple samples were first analyzed by one-way ANOVA followed by multiple comparison analysis by least significant difference post hoc test. *P* < 0.05 was considered to indicate a statistically significant difference.

## Supplementary information

Zhang-Supplementary materials

Original Data File
