# Supplementary material for: Venlafaxine antagonizes the noradrenaline-promoted colon cancer progression by inhibiting the norepinephrine transporter
Source: Cell Death Discov. 2023 May 8;9:152. doi: 10.1038/s41420-023-01447-5 (PMC10167232; doi:10.1038/s41420-023-01447-5)
Supplement: Supplementary file 2 — Original Data File [file 41420_2023_1447_MOESM2_ESM.pdf]

Figure1C

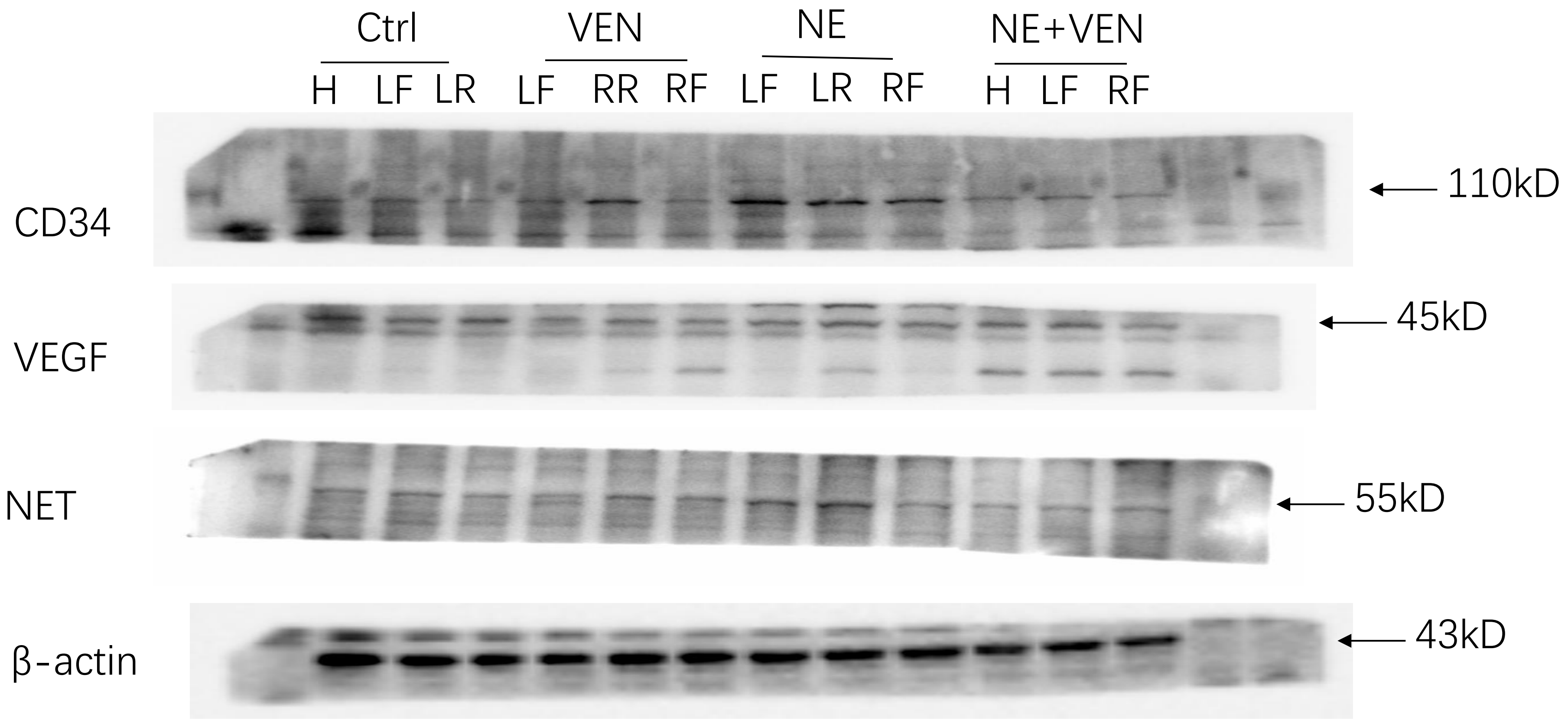

Ctrl
VEN
NE
NE+VEN
H LF LR LF RR RF LF LR RF H LF RF
CD34
110kD
VEGF
45kD
NET
55kD
β-actin
43kD

Figure 2C

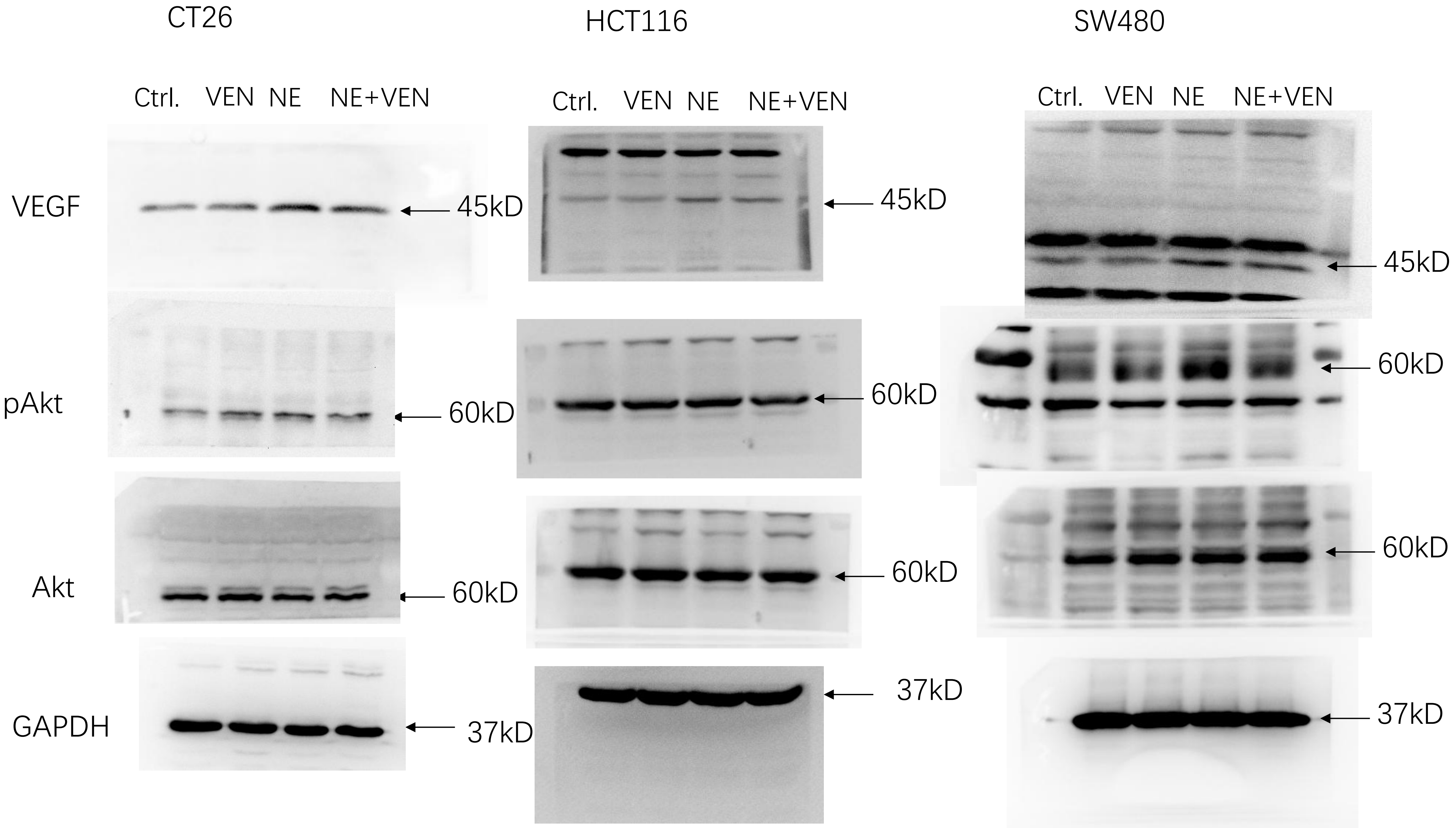
CT26
HCT116
SW480
Ctrl.
VEN
NE
NE+VEN
VEGF
pAkt
Akt
GAPDH
45kD
60kD
60kD
37kD

Figure 3A

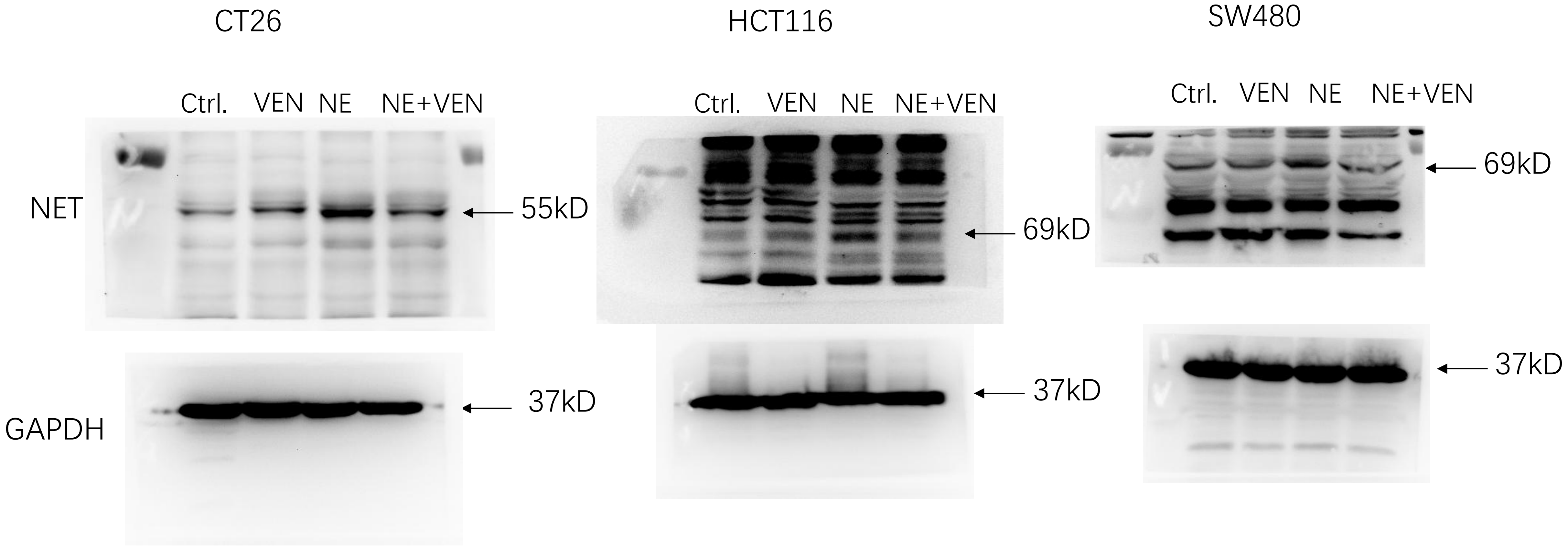

CT26 NET antibody： GTX82626（55kD）

HCT116/SW480 NET antibody： GTX47102（69kD）

The antibody instructions are as follows:

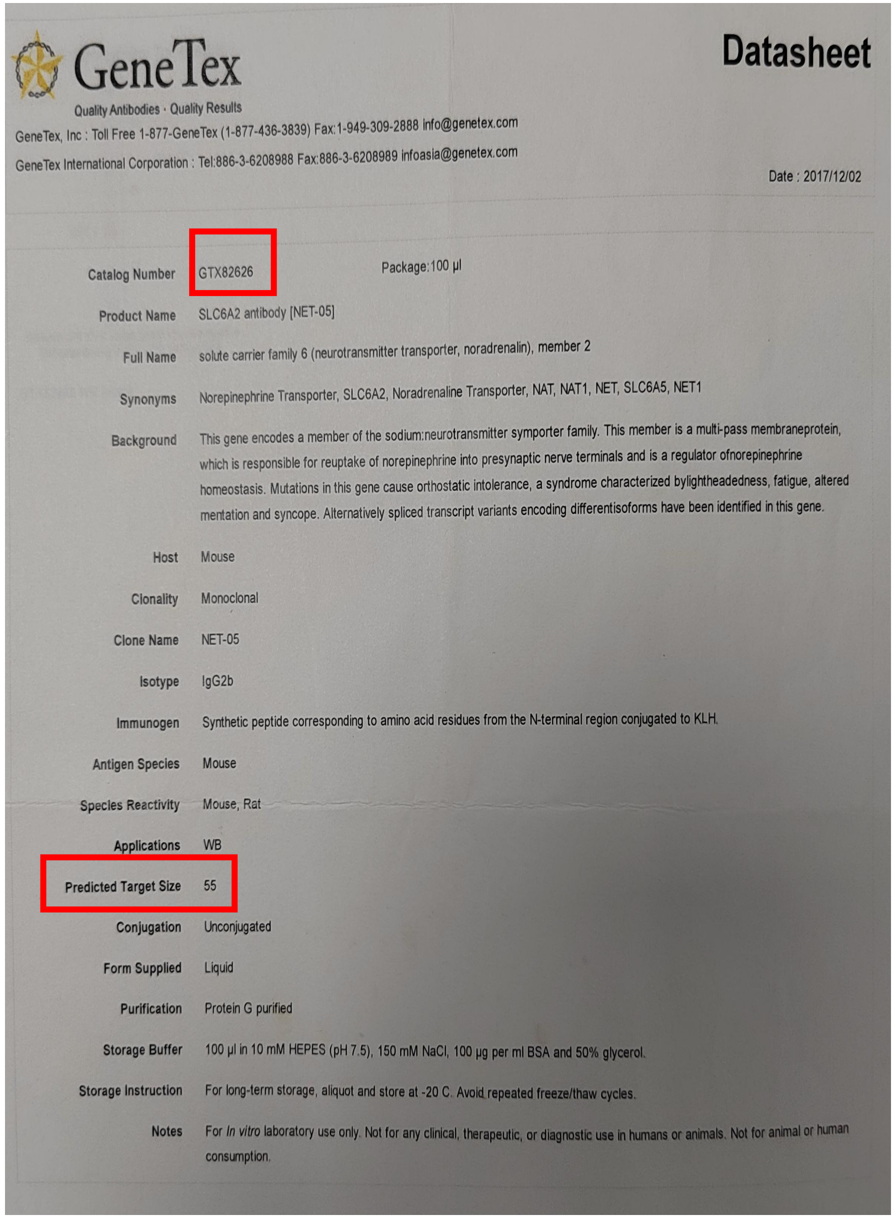

GeneTex

Quality Antibodies · Quality Results

Datasheet

GeneTex, Inc : Toll Free 1-877-GeneTex (1-877-436-3839) Fax:1-949-309-2888 info@genetex.com

GeneTex International Corporation : Tel:886-3-6208988 Fax:886-3-6208989 infoasia@genetex.com

Date : 2017/12/02

Catalog Number	GTX82626 Package:100 µl
Product Name	SLC6A2 antibody [NET-05]
Full Name	solute carrier family 6 (neurotransmitter transporter, noradrenalin), member 2
Synonyms	Norepinephrine Transporter, SLC6A2, Noradrenaline Transporter, NAT, NAT1, NET, SLC6A5, NET1
Background	This gene encodes a member of the sodium:neurotransmitter symporter family. This member is a multi-pass membraneprotein, which is responsible for reuptake of norepinephrine into presynaptic nerve terminals and is a regulator ofnorepinephrine homeostasis. Mutations in this gene cause orthostatic intolerance, a syndrome characterized bylightheadedness, fatigue, altered mentation and syncope. Alternatively spliced transcript variants encoding differentisoforms have been identified in this gene.
Host	Mouse
Clonality	Monoclonal
Clone Name	NET-05
Isotype	IgG2b
Immunogen	Synthetic peptide corresponding to amino acid residues from the N-terminal region conjugated to KLH.
Antigen Species	Mouse
Species Reactivity	Mouse, Rat
Applications	WB
Predicted Target Size	55
Conjugation	Unconjugated
Form Supplied	Liquid
Purification	Protein G purified
Storage Buffer	100 µl in 10 mM HEPES (pH 7.5), 150 mM NaCl, 100 µg per ml BSA and 50% glycerol.
Storage Instruction	For long-term storage, aliquot and store at -20 C. Avoid repeated freeze/thaw cycles.
Notes	For *In vitro* laboratory use only. Not for any clinical, therapeutic, or diagnostic use in humans or animals. Not for animal or human consumption.

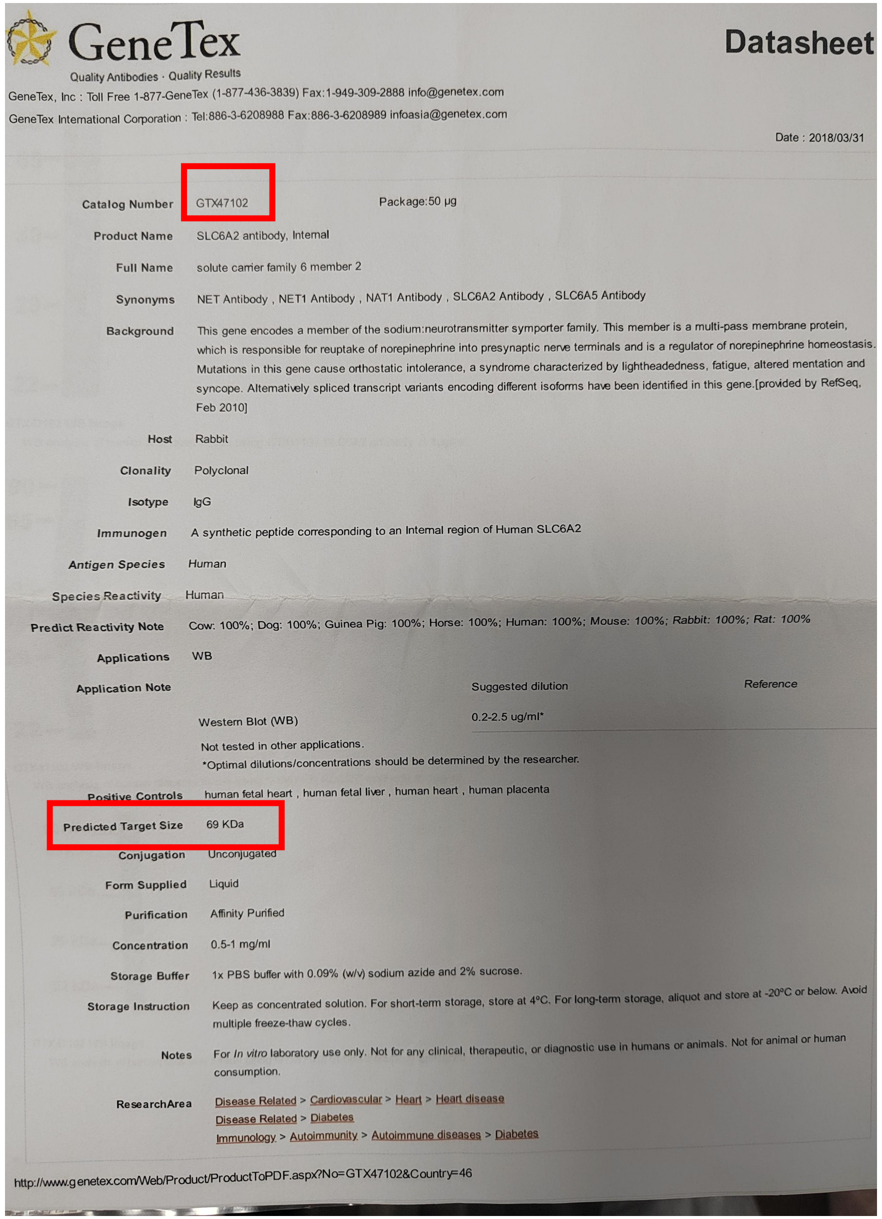

GeneTex

Quality Antibodies · Quality Results

Datasheet

GeneTex, Inc : Toll Free 1-877-GeneTex (1-877-436-3839) Fax:1-949-309-2888 info@genetex.com

GeneTex International Corporation : Tel:886-3-6208988 Fax:886-3-6208989 infoasia@genetex.com

Date : 2018/03/31

Catalog Number	GTX47102 Package:50 µg
Product Name	SLC6A2 antibody, Internal
Full Name	solute carrier family 6 member 2
Synonyms	NET Antibody , NET1 Antibody , NAT1 Antibody , SLC6A2 Antibody , SLC6A5 Antibody
Background	This gene encodes a member of the sodium:neurotransmitter symporter family. This member is a multi-pass membrane protein, which is responsible for reuptake of norepinephrine into presynaptic nerve terminals and is a regulator of norepinephrine homeostasis. Mutations in this gene cause orthostatic intolerance, a syndrome characterized by lightheadedness, fatigue, altered mentation and syncope. Alternatively spliced transcript variants encoding different isoforms have been identified in this gene.[provided by RefSeq, Feb 2010]
Host	Rabbit
Clonality	Polyclonal
Isotype	IgG
Immunogen	A synthetic peptide corresponding to an Internal region of Human SLC6A2
Antigen Species	Human
Species Reactivity	Human
Predict Reactivity Note	Cow: 100%; Dog: 100%; Guinea Pig: 100%; Horse: 100%; Human: 100%; Mouse: 100%; *Rabbit: 100%; Rat: 100%*
Applications	WB
Application Note	Suggested dilution / Reference<br>Western Blot (WB) 0.2-2.5 ug/ml*<br>Not tested in other applications.<br>*Optimal dilutions/concentrations should be determined by the researcher.
Positive Controls	human fetal heart , human fetal liver , human heart , human placenta
Predicted Target Size	69 KDa
Conjugation	Unconjugated
Form Supplied	Liquid
Purification	Affinity Purified
Concentration	0.5-1 mg/ml
Storage Buffer	1x PBS buffer with 0.09% (w/v) sodium azide and 2% sucrose.
Storage Instruction	Keep as concentrated solution. For short-term storage, store at 4°C. For long-term storage, aliquot and store at -20°C or below. Avoid multiple freeze-thaw cycles.
Notes	For *In vitro* laboratory use only. Not for any clinical, therapeutic, or diagnostic use in humans or animals. Not for animal or human consumption.
ResearchArea	Disease Related > Cardiovascular > Heart > Heart disease<br>Disease Related > Diabetes<br>Immunology > Autoimmunity > Autoimmune diseases > Diabetes

http://www.genetex.com/Web/Product/ProductToPDF.aspx?No=GTX47102&Country=46

Figure 3D

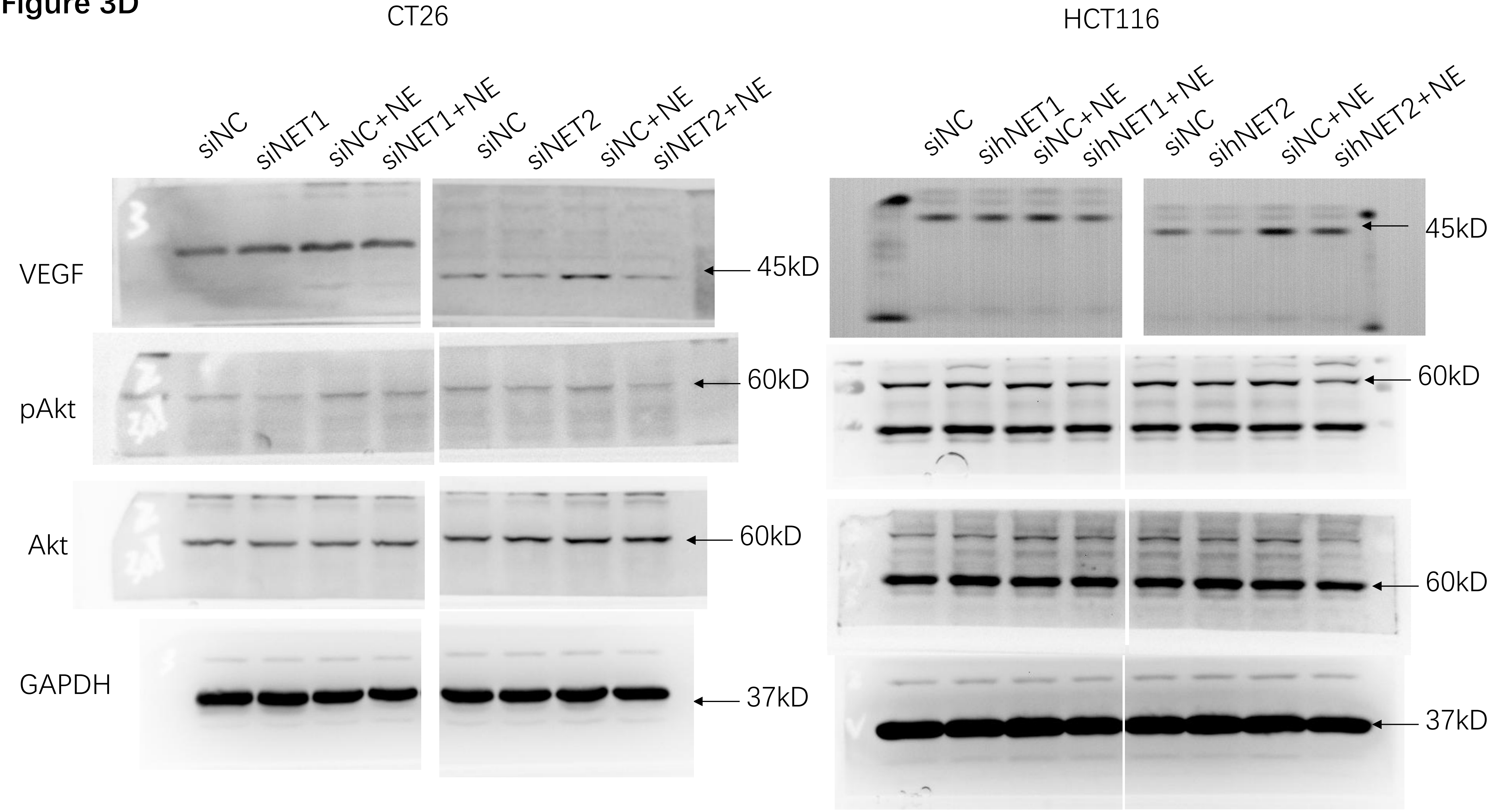
CT26
siNC
siNET1
siNC+NE
siNET1+NE
siNC
siNET2
siNC+NE
siNET2+NE
VEGF
pAkt
Akt
GAPDH
45kD
60kD
60kD
37kD
HCT116
siNC
sihNET1
siNC+NE
sihNET1+NE
siNC
sihNET2
siNC+NE
sihNET2+NE
45kD
60kD
60kD
37kD

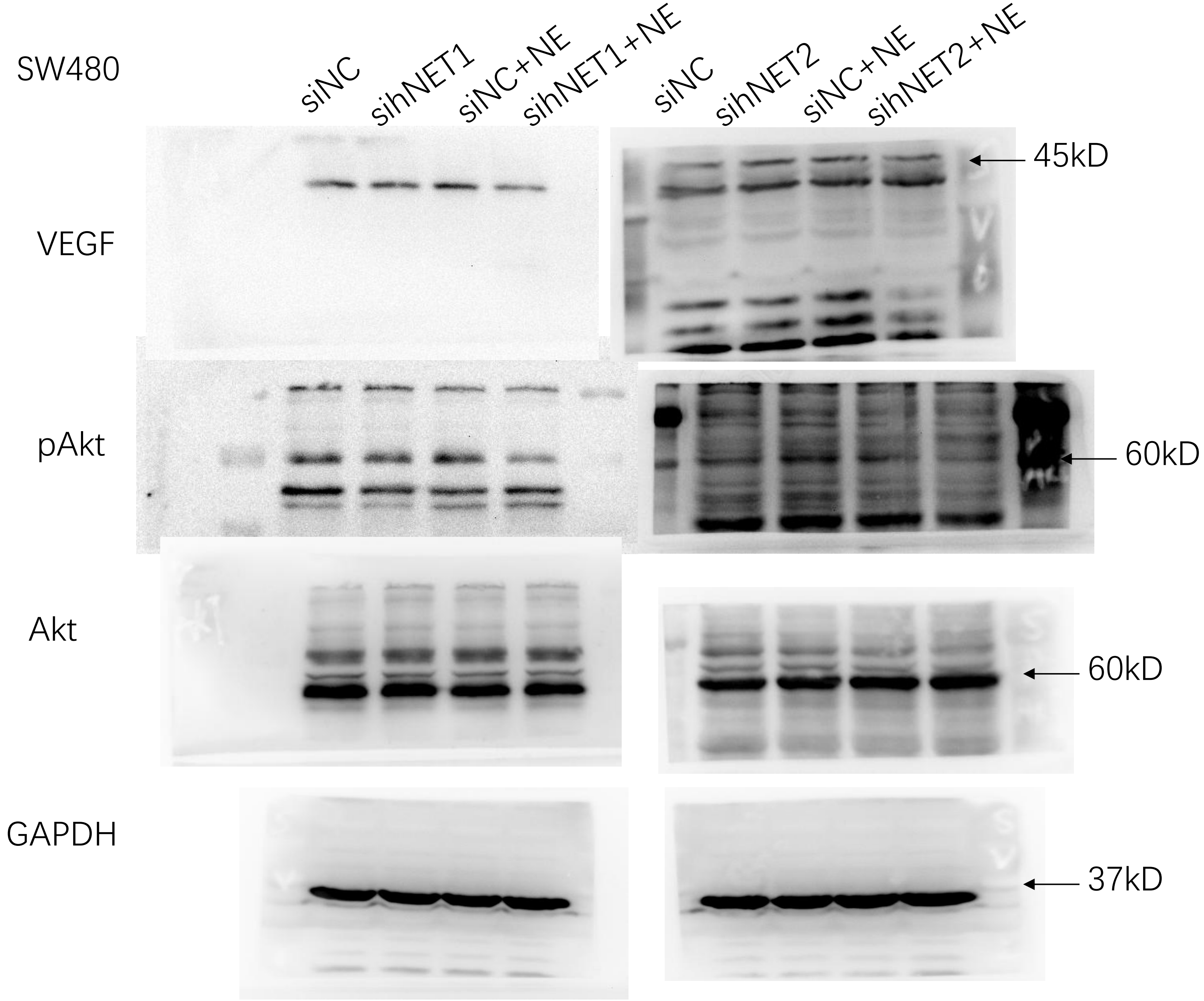
SW480
siNC
sihNET1
siNC+NE
sihNET1+NE
siNC
sihNET2
siNC+NE
sihNET2+NE
VEGF
pAkt
Akt
GAPDH
45kD
60kD
60kD
37kD

Figure 4A

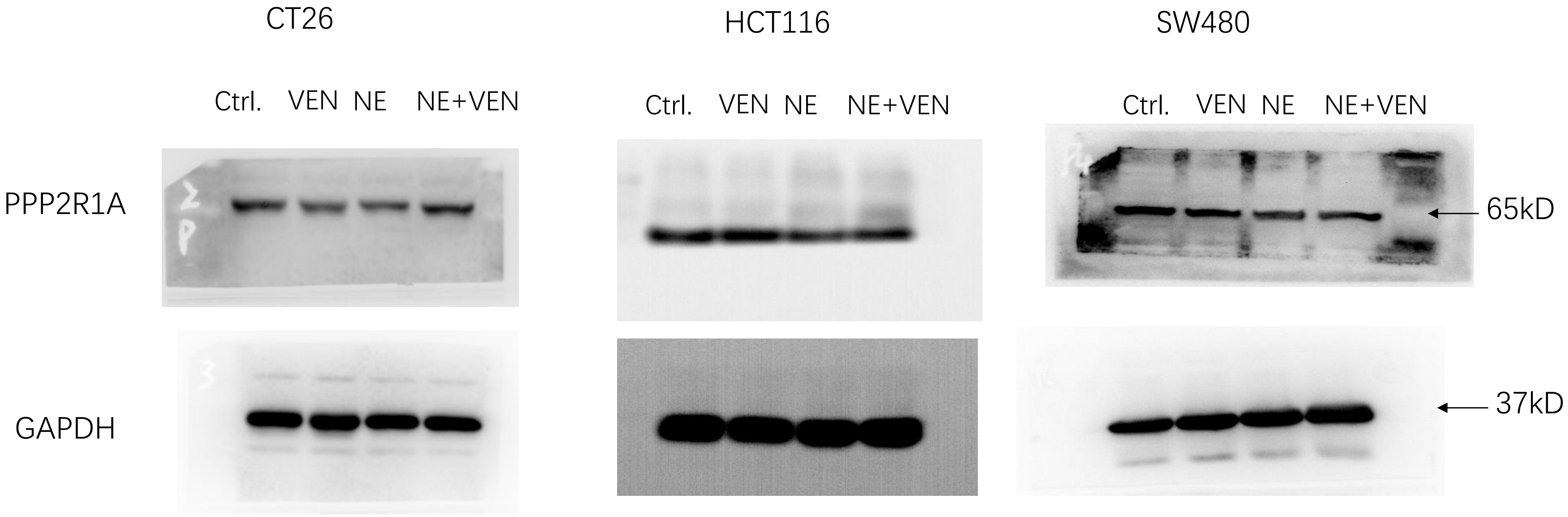

CT26
HCT116
SW480
Ctrl. VEN NE NE+VEN
Ctrl. VEN NE NE+VEN
Ctrl. VEN NE NE+VEN
PPP2R1A
GAPDH
65kD
37kD

Figure 4B

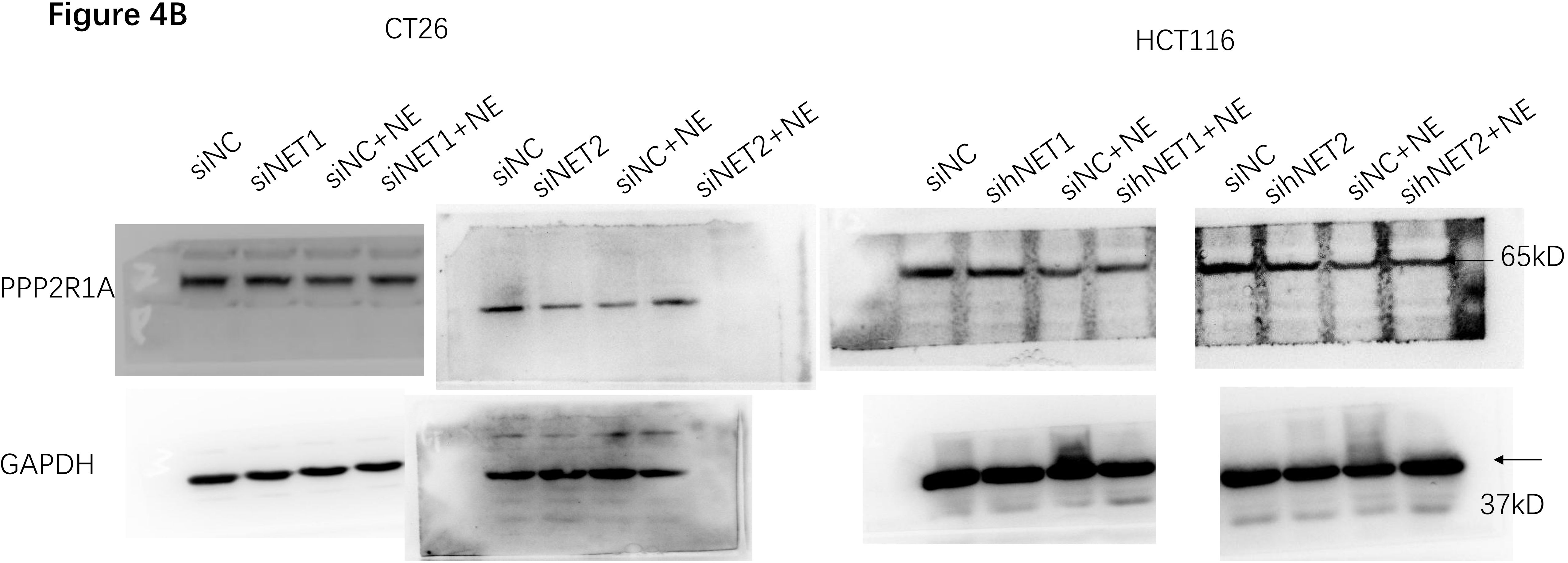
CT26
HCT116
siNC
siNET1
siNC+NE
siNET1+NE
siNC
siNET2
siNC+NE
siNET2+NE
siNC
sihNET1
siNC+NE
sihNET1+NE
siNC
sihNET2
siNC+NE
sihNET2+NE
PPP2R1A
GAPDH
65kD
37kD

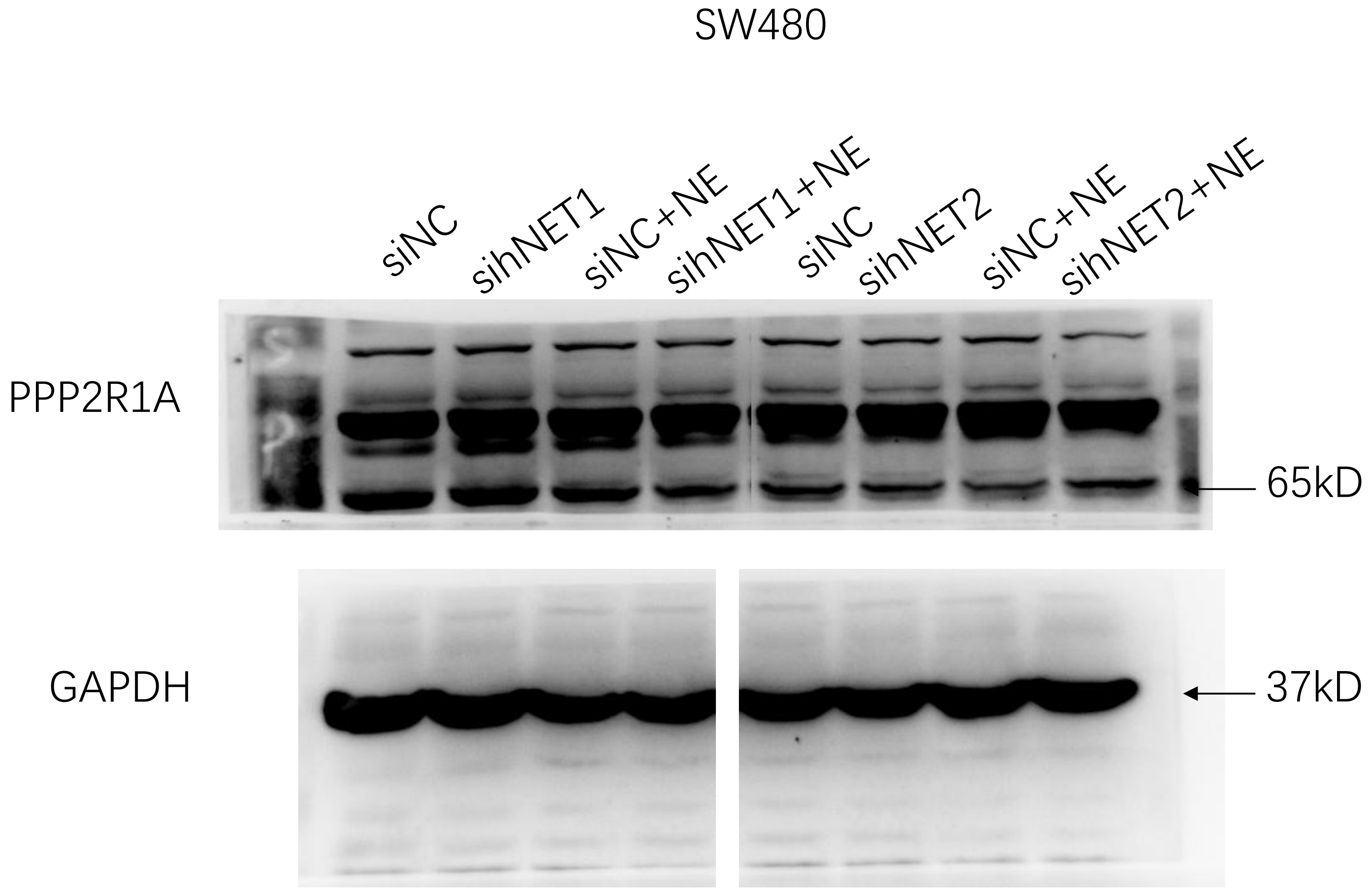
SW480
siNC
sihNET1
siNC+NE
sihNET1+NE
siNC
sihNET2
siNC+NE
sihNET2+NE
PPP2R1A
65kD
GAPDH
37kD

Figure 4C

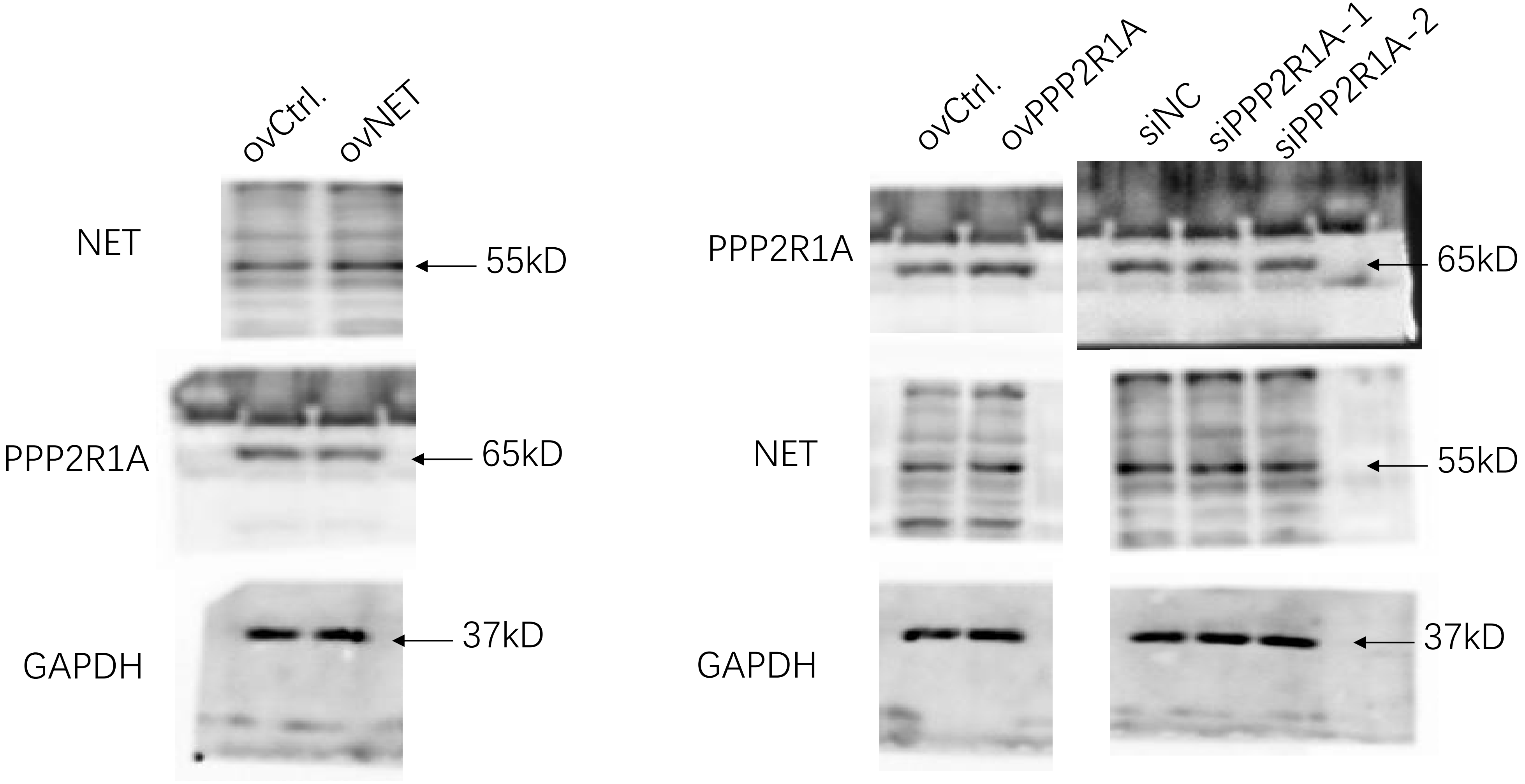
ovCtrl.
ovNET
NET
55kD
PPP2R1A
65kD
GAPDH
37kD
ovCtrl.
ovPPP2R1A
siNC
siPPP2R1A-1
siPPP2R1A-2
PPP2R1A
65kD
NET
55kD
GAPDH
37kD

Figure 4D

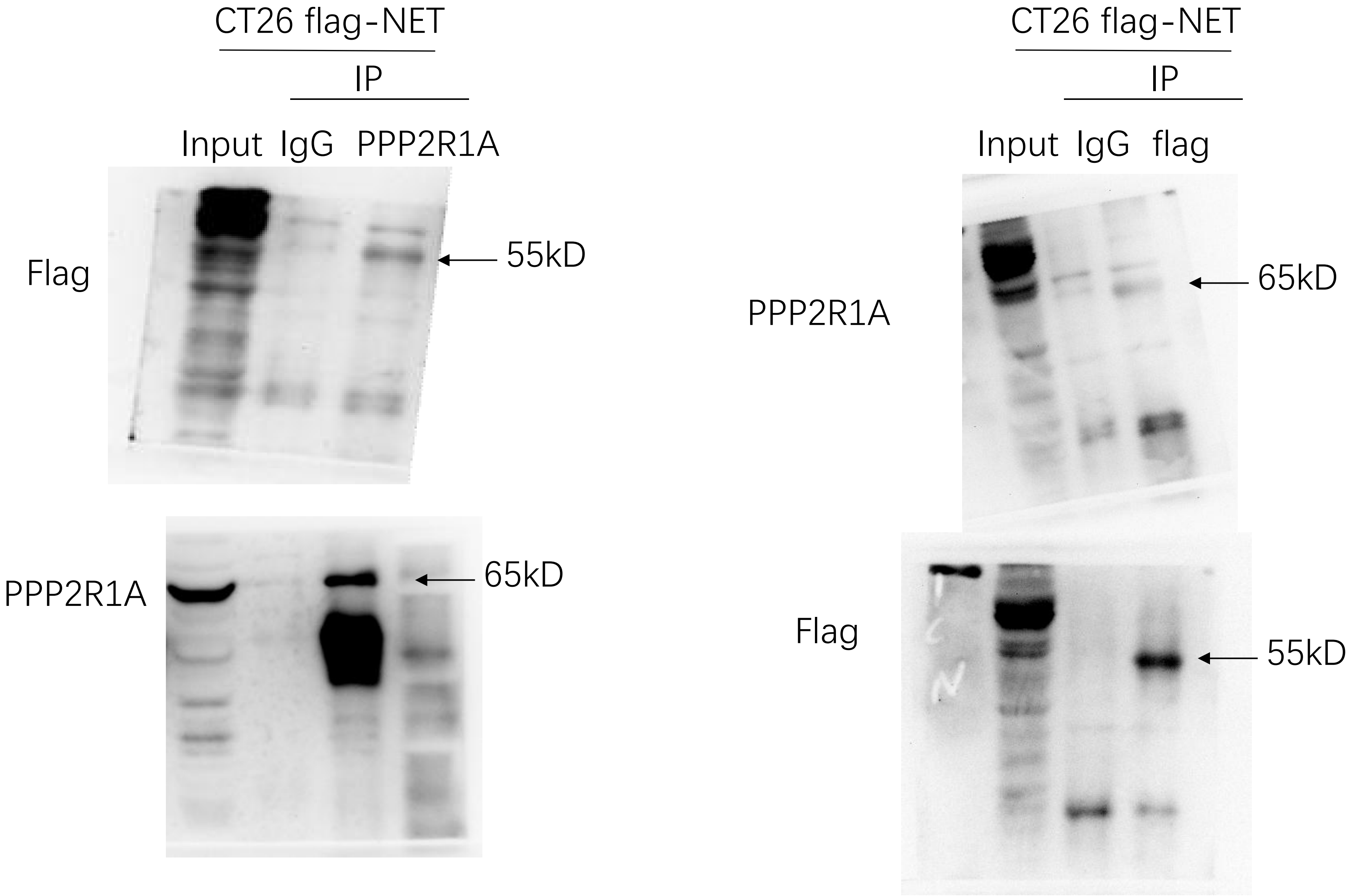
CT26 flag-NET
IP
Input
IgG
PPP2R1A
Flag
55kD
PPP2R1A
65kD
CT26 flag-NET
IP
Input
IgG
flag
PPP2R1A
65kD
Flag
55kD

Figure 5A

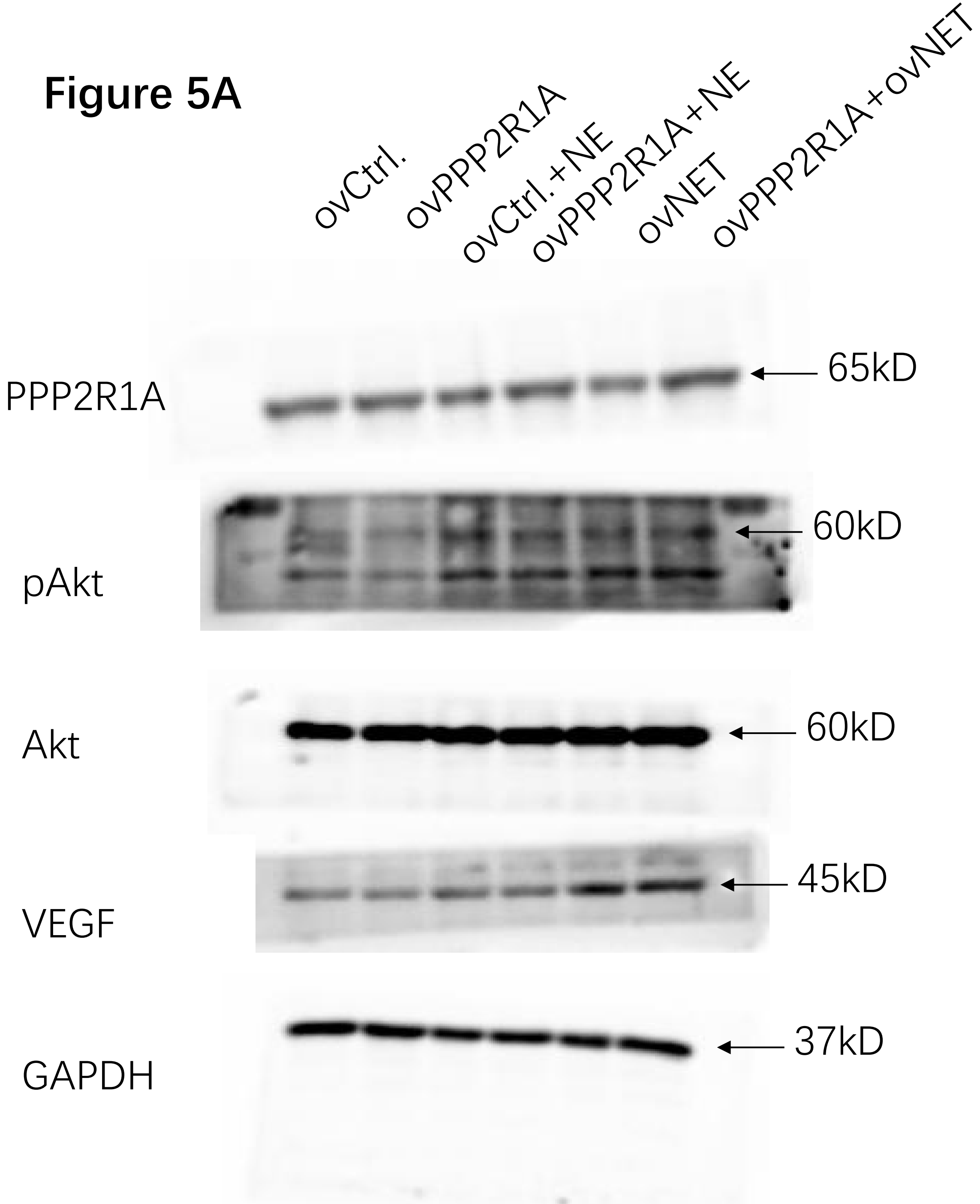

Figure 5B

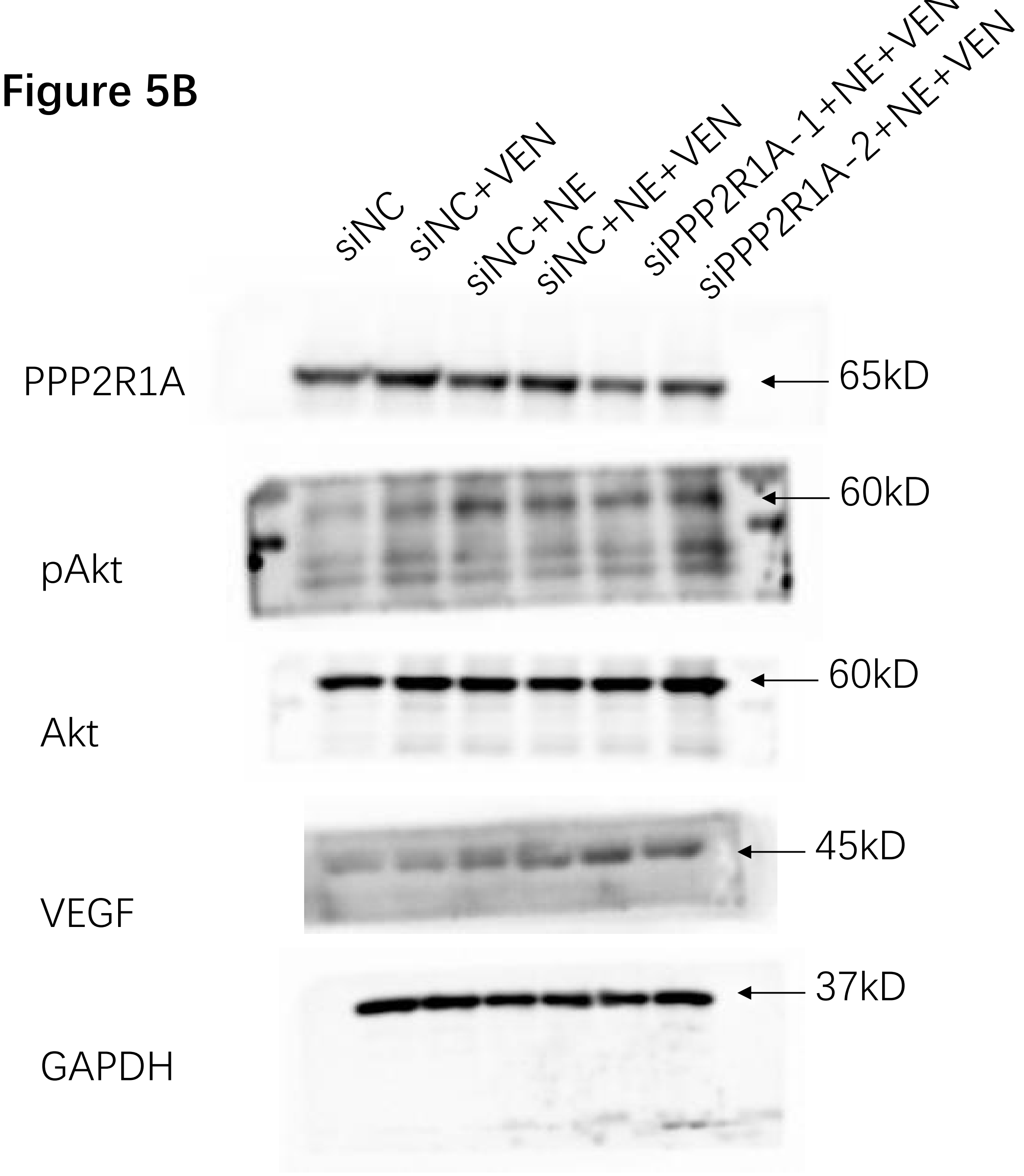

Figure 6C

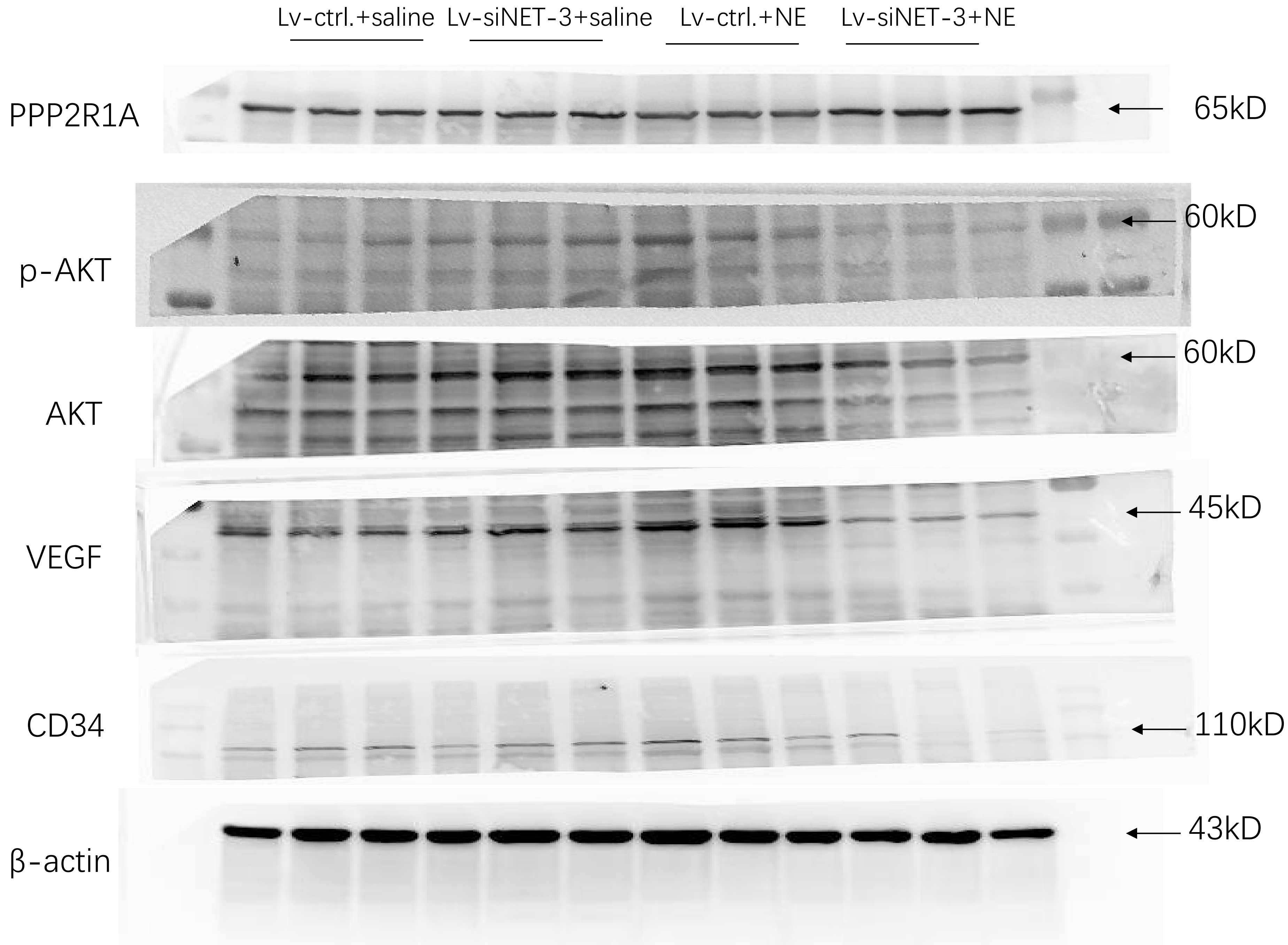
Lv-ctrl.+saline
Lv-siNET-3+saline
Lv-ctrl.+NE
Lv-siNET-3+NE
PPP2R1A
65kD
p-AKT
60kD
AKT
60kD
VEGF
45kD
CD34
110kD
β-actin
43kD

Figure S3C

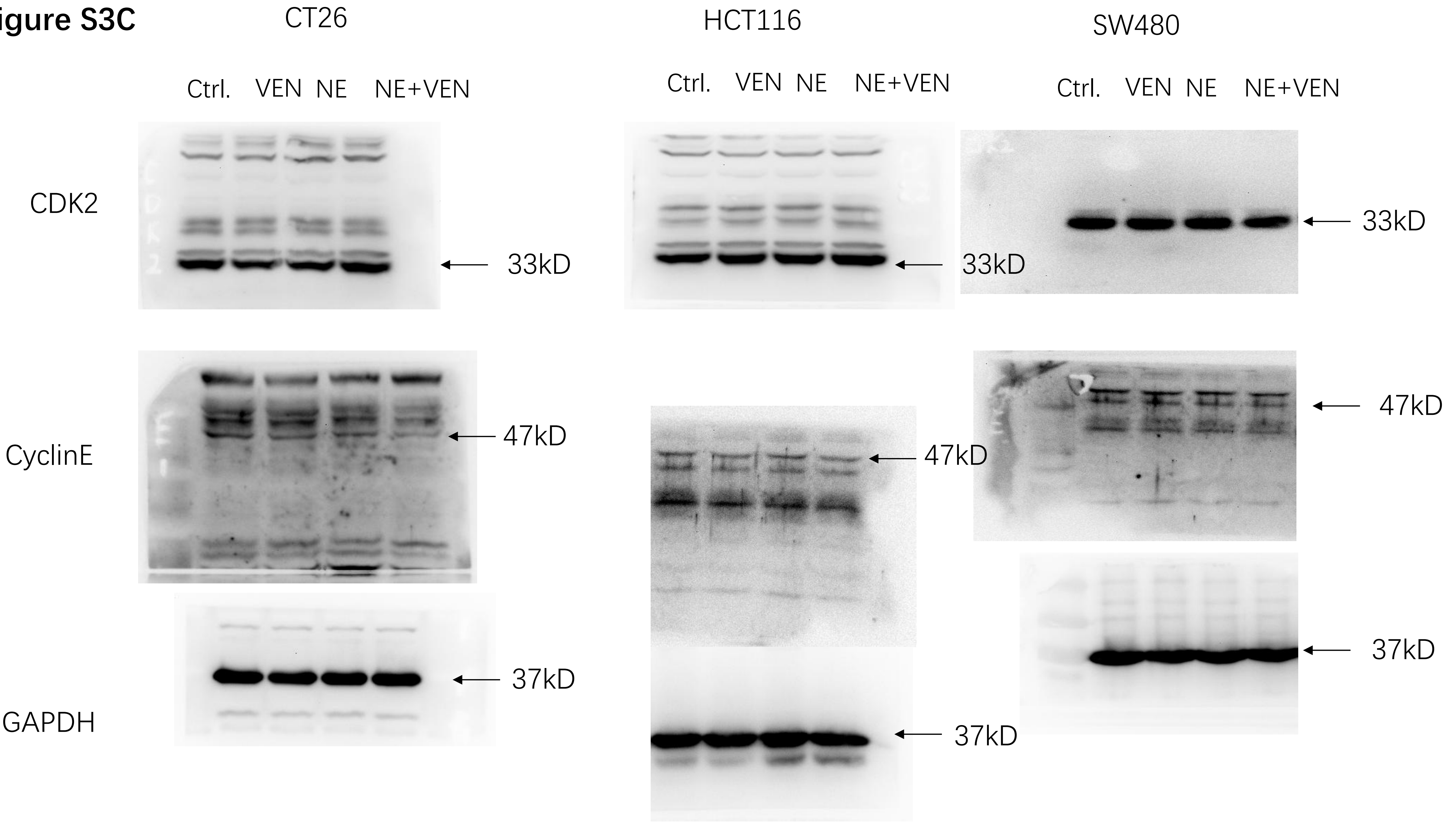
CT26
HCT116
SW480
Ctrl.
VEN
NE
NE+VEN
CDK2
CyclinE
GAPDH
33kD
47kD
37kD

Figure S5A

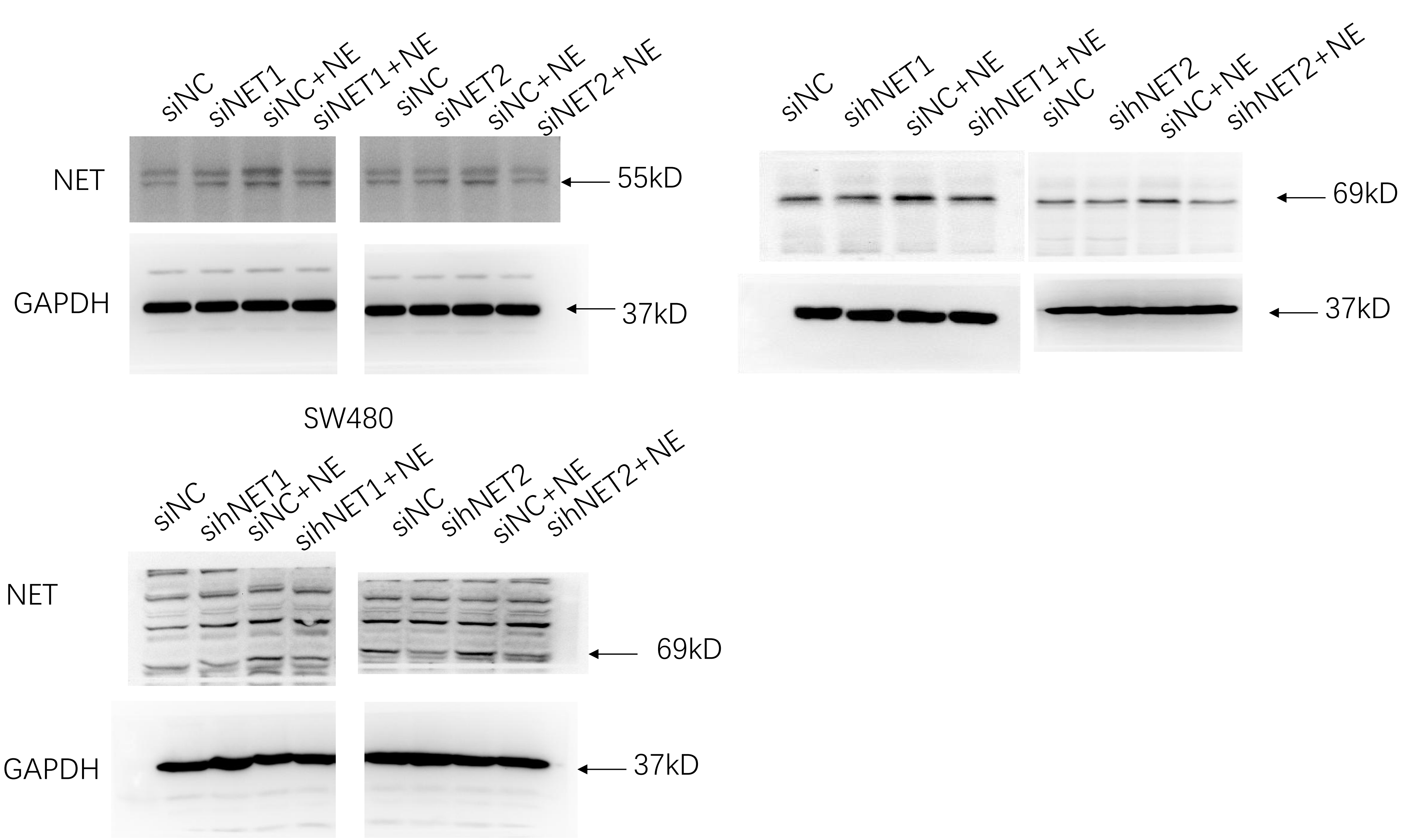
CT26
siNC
siNET1
siNC+NE
siNET1+NE
siNC
siNET2
siNC+NE
siNET2+NE
NET
55kD
GAPDH
37kD
HCT116
siNC
sihNET1
siNC+NE
sihNET1+NE
siNC
sihNET2
siNC+NE
sihNET2+NE
69kD
37kD
SW480
siNC
sihNET1
siNC+NE
sihNET1+NE
siNC
sihNET2
siNC+NE
sihNET2+NE
NET
69kD
GAPDH
37kD

Figure S7

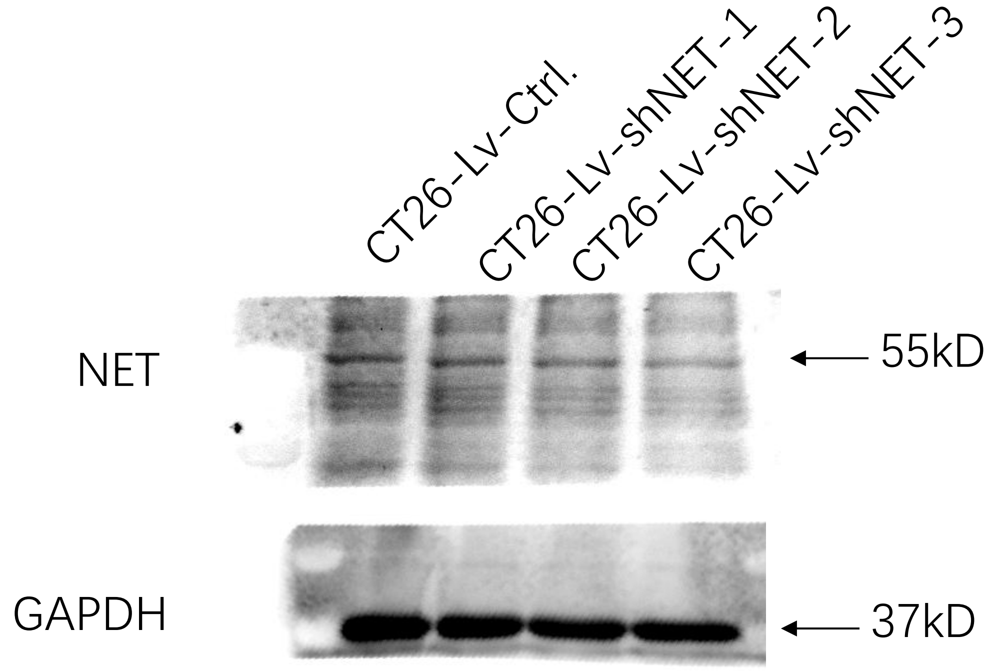
CT26-Lv-Ctrl.
CT26-Lv-shNET-1
CT26-Lv-shNET-2
CT26-Lv-shNET-3
NET
55kD
GAPDH
37kD
