# Supplementary material for: Venlafaxine antagonizes the noradrenaline-promoted colon cancer progression by inhibiting the norepinephrine transporter
Source: Cell Death Discov. 2023 May 8;9:152. doi: 10.1038/s41420-023-01447-5 (PMC10167232; doi:10.1038/s41420-023-01447-5)
Supplement: Supplementary file 1 — Zhang-Supplementary materials [file 41420_2023_1447_MOESM1_ESM.doc]

**Supplementary figures**

**Fig. S1. Adrenergic load is increased in patients with colorectal cancer (CRC).** **A** The serum of patients with CRC and normal subjects were collected. ELISA was performed, which revealed the level of serum NE significantly increased in patients with CRC. **B** The paraffin sections of patients with CRC were analyzed by immunohistochemical analysis of tyrosine hydroxylase (TH). **C** Using the publicly available RNAseq data of CRC gene expression in TCGA database, the expression of TH was analyzed according to tumor topography (T1, T2, T3 and T4), lymph node metastasis (N0, N1 and N2), distant metastasis (M0 and M1) and stages (I, II, III and IV) of patients with CRC. *** *P* < 0.001 in **A**.

**Fig. S2. Norepinephrine (NE) promotes subcutaneous CT26 tumors’ formation *in vivo*.** After 1-week acclimatization, male BALB/c mice were randomly divided into the control group (injected intraperitoneally with saline everyday) and the treatment groups with different concentrations of NE (injected intraperitoneally with NE everyday) and housed for 20 days (n=6 for each group). **A** The survival data showed that 0.125mg/kg of NE was relatively safe to mice. **B** Mice in the control and NE-treatment (injected intraperitoneally with 0.125mg/kg of NE everyday) group were housed for 17 days. The body weights were measured every three days (n=7). **C** Mice in the control and NE-treatment group were housed for 7 days (n=5). Then 1×10^6^ CT26 cells were injected subcutaneously into the right oxters of the mice. For 2 weeks after tumor inoculation, the tumor size was measured. **D** On day 17, the mice were sacrificed and the tumor xenografts were harvested and weighed. Data are expressed as mean ± SD, * *P* < 0.05, ** *P* < 0.01 in **C** and **D**.

**Fig. S3. Norepinephrine (NE) induces an increase of cell population of G2/M phase; venlafaxine (VEN) causes G1-S arrest in colon cancer cells.** Colon cancer cells were treated with control, VEN (10 µM), NE (10 µM), and drug combination [(NE (10 µM) + VEN (10 µM)] for 48h separately. **A** Cell cycle distribution was analyzed by flow cytometry to determine the effect of colon cancer cells treated with NE/VEN. **B** Representative images of flow cytometry of each group. **C** The expression of cyclin E and CDK2 was detected by western blotting. The band intensities of CDK2 and CyclinE relative to GAPDH were quantified and normalized to the Ctrl. group. The quantification graphs of normalized band intensities of different replicates were shown in Fig. S8. Data are expressed as mean ± SD (n=5), * *P* < 0.05, ** *P* < 0.01, *** *P* < 0.001 in **A**.

**Fig. S4. Analysis of NET/5-HTT expression in human CRC using TCGA database.** Using the publicly available RNAseq data of CRC gene expression in TCGA database, the expression patterns of NET **(A)** and 5-HTT **(B)** were analyzed according to tumor topography (T1, T2, T3 and T4), lymph node metastasis (N0, N1 and N2), distant metastasis (M0 and M1) and clinical stages (I, II, III and IV) of patients with CRC. * *P* < 0.05, ** *P* < 0.01, *** *P* < 0.001.

**Fig. S5. The knockdown of norepinephrine transporter (NET) using short interfering RNA against NET in colon cancer cells.** Colon cancer cells were transfected with siNET, sihNET, or siNC separately to knocking down NET expression. After 24 hours, they were treated with 10 µM of NE and incubated further. Western blotting **(A)** and qRT-PCR **(B)** were performed and revealed that NE treatment increased the levels of NET protein and NET mRNA, while siNET/sihNET made them downregulated. The band intensities of NET relative to GAPDH were quantified and normalized to the siNC group. The quantification graphs of normalized band intensities of different replicates were shown in Fig. S8. Data are expressed as mean ± SD (n=5), * *P* < 0.05, ** *P* < 0.01, *** *P* < 0.001 in **B**.

**Fig. S6. There is a protein interaction between NET (SLC6A2) and PPP2R1A according to the STRING analysis.**

**Fig. S7. The cell lines stably expressing down-regulated NET were established and identified by western blotting.** The quantification graphs of normalized band intensities of different replicates were shown in Fig. S8.

Fig. S8. Quantification of western blotting results.Data are expressed as mean ± SD, * *P* < 0.05, ** *P* < 0.01, *** *P* < 0.001 in the all followed graph.

Quantification graph of normalized band intensities of different replicates in Fig 2C.

Quantification graph of normalized band intensities of different replicates in Fig 3A.

Quantification graph of normalized band intensities of different replicates in Fig 3D.

Quantification graph of normalized band intensities of different replicates in Fig 4A.

Quantification graph of normalized band intensities of different replicates in Fig 4B.

Quantification graph of normalized band intensities of different replicates in Fig 4C.

Quantification graph of normalized band intensities of different replicates in Fig 5A，5B.

Quantification graph of normalized band intensities of different replicates in Fig S3C.

Quantification graph of normalized band intensities of different replicates in Fig S5A.

Quantification graph of normalized band intensities of different replicates in Fig S7.

**Supplementary tables**

**Supplementary table 1. The antibodies used in this study.**

| Antibody (Catalog) | Dilution | Company |
| --- | --- | --- |
| CD34 (14486-1-AP) | 1:200 (IHC) 1:1000 (WB) | Proteintech Group |
| VEGF (19003-1-AP) | 1:1000 | Proteintech Group |
| NET (GTX82626) | 1:1000 | GeneTex |
| NET (GTX47102) | 1:1000 | GeneTex |
| CDK2 (10122-1-AP) | 1:1000 | Proteintech Group |
| CyclinE1 (11554-1-AP) | 1:1000 | Proteintech Group |
| AKT (10176-2-AP) | 1:1000 | Proteintech Group |
| p-AKT (Ser473) (4060) | 1:1000 | Cell Signaling Technology |
| p-AKT (Thr308) (13038) | 1:1000 | Cell Signaling Technology |
| PPP2R1A (15882-1-AP) | 1:1000 | Proteintech Group |
| β-actin (sc-47778) | 1:1000 | Santa Cruz Biotechnology |
| GAPDH（sc‑32233） | 1:1000 | Santa Cruz Biotechnology |
| Anti-mouse secondary antibody（115-035-146） | 1:5000 | Jackson ImmunoResearch Laboratories |
| Anti-rabbit secondary antibody（111-035-144） | 1:5000 | Jackson ImmunoResearch Laboratories |

**Supplementary table 2. Oligonucleotides used in this study.**

| Name | Sequences (5'→3') |
| --- | --- |
| siNC sense | UUCUCCGAACGUGUCACGUTT |
| siNC antisense | ACGUGACACGUUCGGAGAATT |
| siNET1 sense | CCAUACCAAAUACUCCAAATT |
| siNET1 antisense | UUUGGAGUAUUUGGUAUGGTT |
| siNET2 sense | GGACACAUCCUAUUGAAAATT |
| siNET2 antisense | UUUUCAAUAGGAUGUGUCCTT |
| siPPP2R1A-1 sense | GUCCGACUGAAUAUCAUCUTT |
| siPPP2R1A-1 antisense | AGAUGAUAUUCAGUCGGACTT |
| siPPP2R1A-2 sense | CUAGCUGAAGAUGCCAAGUTT |
| siPPP2R1A-2 antisense | ACUUGGCAUCUUCAGCUAGTT |
| sihNET1 sense | GAUUUCGUGACUGUAGUUUTT |
| sihNET1 antisense | AAACUACAGUCACGAAAUCTT |
| sihNET2 sense | GGAGAAGGAGAGCUACCAATT |
| sihNET2 antisense | UUGGUAGCUCUCCUUCUCCTT |
|  |  |

**Supplementary table 3. Primers used in this study.**

| Name | Primers Sequences (5'→3') |
| --- | --- |
| GAPDH Forward | GGAGAGTGTTTCCTCGTCCC |
| GAPDH Reverse | ACTGTGCCGTTGAATTTGCC |
| NET Forward | GCGGTTCCCTTATCTCTGCT |
| NET Reverse | CAGCGTGTATGGAATCAGGA |
| VEGF Forward | TACTGCCGTCCGATTGAGACC |
| VEGF Reverse | CACAGTGAACGCTCCAGGATTTA |
| PPP2R1A Forward | CTTCCTGCCATCGTGGAACT |
| PPP2R1A Reverse | ACTCTGCAGGGTGCTGTTAT |
| hGAPDH Forward | GTAGAGGCAGGGATGATGTTC |
| hGAPDH Reverse | GCCAAAAGGGTCATCATCTC |
| hNET Forward | GCGCTCATCCCAGTGTCTAA |
| hNET Reverse | GGATCAAGAAGGCACCGCC |
| hVEGF Forward | ATGCGGATCAAACCTCACCA |
| hVEGF Reverse | CACCAACGTACACGCTCCAG |
| hPPP2R1A Forward | TCGACCAATGGCAAATCGGA |
| hPPP2R1A Reverse | CTGTTGAGGCGAAGCTGAAC |
